# Probing surface Earth reactive silica cycling using stable Si isotopes: Mass balance, fluxes, and deep time implications

**DOI:** 10.1126/sciadv.adi2440

**Published:** 2023-12-06

**Authors:** Shaily Rahman, Elizabeth J. Trower

**Affiliations:** ^1^ Department of Geological Sciences, University of Colorado Boulder, Boulder, CO, USA.; ^2^ Institute of Arctic and Alpine Research (INSTAAR), University of Colorado Boulder, Boulder, CO, USA.

## Abstract

Geological reservoir δ^30^Si values have increasingly been applied as paleoclimate and paleoproductivity proxies. Many of these applications rely on the assumption that the surface Earth Si isotope budget is in mass balance with bulk silicate Earth, such that trends in δ^30^Si over time can be attributed to changes in flux to or from key silica reservoirs. We compiled δ^30^Si data from modern reservoirs representing the major sources and sinks of surface Earth reactive silica, to which we applied an inverse model to test assumptions about mass balance. We found that δ^30^Si values of reverse weathering products must closely match those of diatoms, conflicting with previous assumptions. Model results also revealed that of the 19 to 21 teramoles per year Si released during silicate mineral weathering, ~10 to 18 teramoles per year is stored in terrestrial silica sinks, consistent with assumptions of incongruent weathering reactions. Our results demonstrated that the modern silica cycle summary is in isotopic mass balance.

## Teaser

Inverse modeling of δ^30^Si values of surface Earth reactive silica informs weathering stoichiometry and utility as a proxy.

## INTRODUCTION

Despite its abundance in the Earth’s crust, there are relatively few tools that directly measure the reactions and transformations of Si through its various reactive pools for either the modern or ancient earth. In addition to geomorphic processes such as tectonic uplift (*1*), reactive Si transport through various reservoirs of the surface Earth (e.g., global oceans, biosphere, rocks and sediment, and cryosphere) applies critical controls on the evolution of the atmosphere and ocean chemistry through Earth’s history, both in present-day cycles and in Earth’s past. The development of new analytical capabilities in the past ~25 years has led to accurate quantification of stable Si isotope abundances (e.g., ^29^Si and ^30^Si). We can now measure nuances in the silica cycle that we were unable to measure before, such as nonconservative behavior in estuaries (*2*), dissolution and precipitation reactions in subterranean estuaries (*3*, *4*), dissolution and authigenic clay formation in marine porewater (*5*), pedogenesis and mineral formation in soils (*6*), silica utilization and paleoproductivity in the ocean (*7*, *8*), chert formation (*9*), and serpentinization in the seafloor (*10*). Implicit within the interpretation of stable Si isotope compositions of surface earth processes, particularly applied to deep time, is that the reactive Si cycle is in isotopic mass balance with the bulk silicate Earth (BSE). However, the conceptualization of the modern surface Earth reactive silica cycle has undergone major changes within the last 20 years, which is about the same time frame in which the application of stable Si isotopes has proliferated. Often, summaries of the marine Si budget are used to establish mass balance (*7*, *11*). Changes in the most recent marine silica budget include a >50% increase in input fluxes, an increase of >30% in output fluxes, and a reduced estimate of Si residence time by ~2-fold in the ocean (Table 1) (*12*). As a community, we have not yet assessed whether the modern silica budget is in Si isotopic mass balance, or whether these substantial updates to the silica budget have affected interpretations of proxy reconstructions with respect to isotopic mass balance.

**Table 1.** Compilation of major summaries of the marine silica cycle in the last ~30 years. Flux estimates from the summaries (*12*, *34*, *41*, *65*, *66*) along with mean δ^30^Si values for each input and output parameter are also included (see table S1). n.d., not determined; LT = low temperature.

	1995 Budget (*41*)	2002 Budget (*65*)	2013 Budget (*34*)	2019 Budget (*66*)	2021 Budget (*12*)	Mean δ^30^Si (‰)	N*
**Inputs (Tmol/year)**							
River (dSi)	5.0 ± 1.1	5.6	6.2 ± 1.8	6.3 ± 1.8	6.2 ± 1.8	1.21 ± 0.76	372
River (aSi)	n.d.	n.d.	1.1 ± 0.2	0.2 ± 0.5	2.1 ± 1.0	−0.27 ± 0.30	75
Fresh groundwater	n.d.	n.d.	0.6 ± 0.6	0.6–3.8 (± 2.0)	0.7	1.33 ± 0.63	21
Marine groundwater	n.d.	n.d.	n.d.		1.6	1.69 ± 0.95	10
Eolian	0.5 ± 0.5	0.5	0.5 ± 0.5	0.05 ± 0.05	0.5 ± 0.5	−1.03 ± 0.70	194
LT dissolution of siliceous minerals and sediment	0.4 ± 0.3	n.d.	1.9 ± 0.7	0.1 ± 0.2	1.9 ± 0.7	−0.3	
Hydrothermal inputs (high + low temperature)	0.2 ± 0.1	0.6	0.6 ± 0.4	0.4 ± 0.2	1.7 ± 0.8	−0.3 ± 0.14	2
Ice sheet meltwater	n.d.	n.d.	n.d.	n.d.	0.33 ± 0.26	0.25 ± 0.37	40
**Outputs (Tmol/year)**							
Coastal diatom burial	1.2 ± 0.7	2.4–3.1	3.3 ± 2.1	3.9–6.2 (± 1.5)	3.7 ± 2.1	1.13 ± 0.44†	50
Deep sea diatom burial	5.9 ± 1.1	4.1–4.3	3.0 ± 1.2	3.5 ± 1.0	5.5 ± 1.2	1.13 ± 0.44†	50
Reverse weathering	n.d.	n.d.	1.5 ± 0.5	0	4.7 ± 2.3		
Sponge burial	n.d.	n.d.	3.6 ± 3.7	0.02–0.9 (± 0.9)	1.7 ± 1.6	−1.95 ± 1.48	72

*Number of δ^30^Si measurements.

†All diatom data are pooled into a combined distribution in the model referred to later in the text as δ^30^Si_Diatom_.

Mass balance is a prerequisite to interpreting reactive Si transport processes over the surface earth, including processes critical to C cycling, across geological time. For example, a principal control on Earth’s atmospheric CO_2_ concentrations over long time scales is thought to be chemical weathering of terrestrial silicate rocks (*13*, *14*) by carbonic acid. Atmospheric CO_2_ is consumed, releasing dissolved silica (dSi); dissolved inorganic carbon, mainly in the form of HCO_3_^−^; cations; and a host of trace elements to solution. The feedback of silicate weathering on atmospheric CO_2_ concentrations is often modeled by applying a stoichiometric relationship between Si and C (*15*) dependent on an assumption of complete (congruent) or incomplete (incongruent) dissolution of the primary mineral phase. Typically, congruent weathering reactions of silicates of the upper continental crust are represented by the complete dissolution of wollastonite (CaSiO_3_) (*16*)

$$2{\mathrm{CO}}_{2}+3H_{2}O+{\mathrm{Ca}\mathrm{SiO}}_{3}\to {\mathrm{Ca}}^{2+}+2{{H\mathrm{CO}}_{3}}^{-}+H_{4}{\mathrm{SiO}}_{4}$$ (1)

The stoichiometry of dissolved Si to CO_2_ of this equation is 0.5 mol/mol. Of the 2 moles of bicarbonate that is produced, it is typically assumed that 1 mole is consumed by the formation of calcium carbonate

$${\mathrm{Ca}}^{2+}+2{{H\mathrm{CO}}_{3}}^{-}\to {\mathrm{Ca}\mathrm{CO}}_{3}+{\mathrm{CO}}_{2}+H_{2}O$$ (2)

such that in the net silicate weathering reaction, 1 mole of CO_2_ reacts to form 1 mole of dSi (H_4_SiO_4_)

$${\mathrm{CO}}_{2}+2H_{2}O+{\mathrm{CaSiO}}_{3}\to {\mathrm{CaCO}}_{3}+H_{4}{\mathrm{SiO}}_{4}$$ (3)

Unlike congruent weathering, incongruent reactions involve the formation of secondary silicate mineral phases as a product of CO_2_ consumption; in other words, some of the dSi released by weathering is incorporated back into a mineral phase. The stoichiometry of Si to C in incongruent silicate weathering reactions can be represented by the incomplete dissolution of the aluminosilicate K-spar (KAlSi_3_O_8_)

$$2{K\mathrm{AlSi}}_{3}O_{8}+2{\mathrm{CO}}_{2}+11H_{2}O\to {\mathrm{Al}}_{2}{\mathrm{Si}}_{2}O_{5}{\left(\mathrm{OH}\right)}_{4}+2\;K^{+}+2{{H\mathrm{CO}}_{3}}^{-}+4H_{4}{\mathrm{Si}O}_{4}$$ (4)

whereby 2 moles of dSi are produced per 1 mole of CO_2_ consumed. Another example of an incongruent weathering reaction where Na-rich plagioclase (“An_20_”) is altered to halloysite that has been used recently is as follows (*17*)

$$1.67{\mathrm{An}}_{20}+3H_{2}O+2{\mathrm{CO}}_{2(\mathrm{aq})}\to \mathrm{Halloysite}+1.33{\mathrm{Na}}^{+}+2.67{\mathrm{SiO}}_{2(\mathrm{aq})}+0.33{\mathrm{Ca}}^{2+}+2{{H\mathrm{CO}}_{3}}^{-}$$ (5)

In Eq. 5, the stoichiometric ratio of Si:CO_2_ is 1.335 mol/mol. For incongruent weathering, some of the alkalinity that is released is also consumed by the formation of CaCO_3_, which rereleases CO_2_ to the atmosphere and changes the net weathering reaction stoichiometry of Si:CO_2_. In our subsequent analyses, we consider only the stoichiometry of the initial weathering reaction (i.e., Eqs. 1, 4, and 5) and not carbonate formation. Whether these reactions are thermodynamically or kinetically controlled depends on the time scales used in each modeling study (*18*–*20*). Constraining the stoichiometry of the reaction (i.e., weathering congruency) and the way that it is modeled on a global scale is fundamental to evaluating the effectiveness of the silicate mineral weathering feedback on atmospheric CO_2_ concentrations and climate evolution.

Dissolved constituents resulting from silicate mineral weathering reactions are delivered to rivers and subterranean estuaries via surface runoff and percolation. River and groundwater discharge are, in turn, the principal vectors through which dSi is delivered to the surface ocean (*12*). Dissolved Si is removed from the water column via silica biomineralization (e.g., formation of diatom frustules and sponge spicules) and sinking of silica biominerals, or opal, to the seafloor. In canonical conceptual models, most of this material is assumed to dissolve in the top 20 cm of the sediment column and diffuse back into the overlying water column (*21*). It is further assumed that the biogenic opal that escapes dissolution is buried as unaltered amorphous opaline phases, which eventually undergo deep diagenesis over long time scales to eventually form chert precursor phases (opal-CT) and chert (*21*). Recently, a second mode of silica removal from the ocean has been verified as a substantial reaction pathway: reverse weathering, defined as diagenetic alteration of biogenic silica at the sediment-water interface to an aluminosilicate phase (*22*–*26*).

In 1966, reverse weathering reactions were formally defined as a suite of reactions which had to occur in the ocean to maintain the mass balance of alkalinity, atmospheric CO_2_, and dSi in the ocean (*27*). Evoking this pathway, some recent studies have attempted to explicitly couple or model the Si and C cycles over geological time scales (*28*–*30*) via reverse weathering reactions. They build upon the attempts in the 1950s and 1960s to balance several marine elemental budgets (*31*) and the formal hypothesis of reverse weathering (*27*). Some of these studies used mass budgets of reactive silica pools across the surface earth based on outdated marine summaries (e.g., summaries that have no fluxes associated with submarine groundwater discharge, sponge spicule burial, or reverse weathering), with assumed stoichiometries of Si-C couplings (e.g., ~1 to 4 mol/mol [HCO_3_^−^]/[Si] consumption ratios during reverse weathering reactions), and approached the evolution of Earth’s climate by modeling C cycle summaries (*29*, *30*). The assumed stoichiometry of dSi to alkalinity consumption of reverse weathering reactions in some of these studies differs considerably from observations, where instead of 1 to 4 mol of bicarbonate ions consumed per mol of dSi, only ~0.14 to 0.28 mol of bicarbonate ion is consumed per mol of dSi (*32*, *33*). Reconciling these differences will be critical to constructing accurate global models of coupled Si-C cycles.

A fundamental first step when using silica summaries in climate reconstructions is to verify mass balance over the surface Earth. One method to test whether the summaries presented in Table 1 are accurate is to determine whether each summary’s respective fluxes are in isotopic mass balance. Stable Si isotopic compositions of modern terrestrial and marine silica pools have been compiled to probe the silica mass balance of the Anthropocene (*7*, *11*) using fluxes in a 2013 marine silica budget (*34*). Since 2013, compilation studies and studies involving multiple proxies, stable, and radiogenic isotope datasets have helped to constrain and revise dSi fluxes from submarine groundwater discharge (*35*, *36*), low-temperature dissolution of river suspended amorphous silica (aSi) phases upon estuarine mixing (*7*), subpolar glacier inputs (*12*), sponge burial (*37*), deep-sea diatom burial, and hydrothermal heat flux (*12*). Incorporation of these changes to mass balance summaries has led to a 40 to 50% increase in both inputs and outputs of dSi from the global ocean, resulting in a revised estimate of oceanic mean residence time from 12.5 to 7.7 ka (*12*). Here, we apply inverse modeling of available marine and terrestrial stable Si isotope records of reactive silica pools in the upper continental crust, starting with the most recent summary (*12*), to answer several questions about the modern silica cycle. Inverse modeling of available stable Si isotope rock/chert/clay records was used to model sources and sinks in the Precambrian silica cycle (*38*). Using a compilation of Si isotopic compositions of modern reactive Si reservoirs, we ask whether marine summaries of the silica cycle (i) are balanced with respect to fluxes and isotopic compositions, (ii) are at steady state, (iii) support congruent or incongruent terrestrial silicate mineral weathering stoichiometries, and (iv) can be used to estimate magnitudes of terrestrial and marine secondary mineral and biogenic silica sinks.

## RESULTS

### Data compilation

**Fig. 1.**
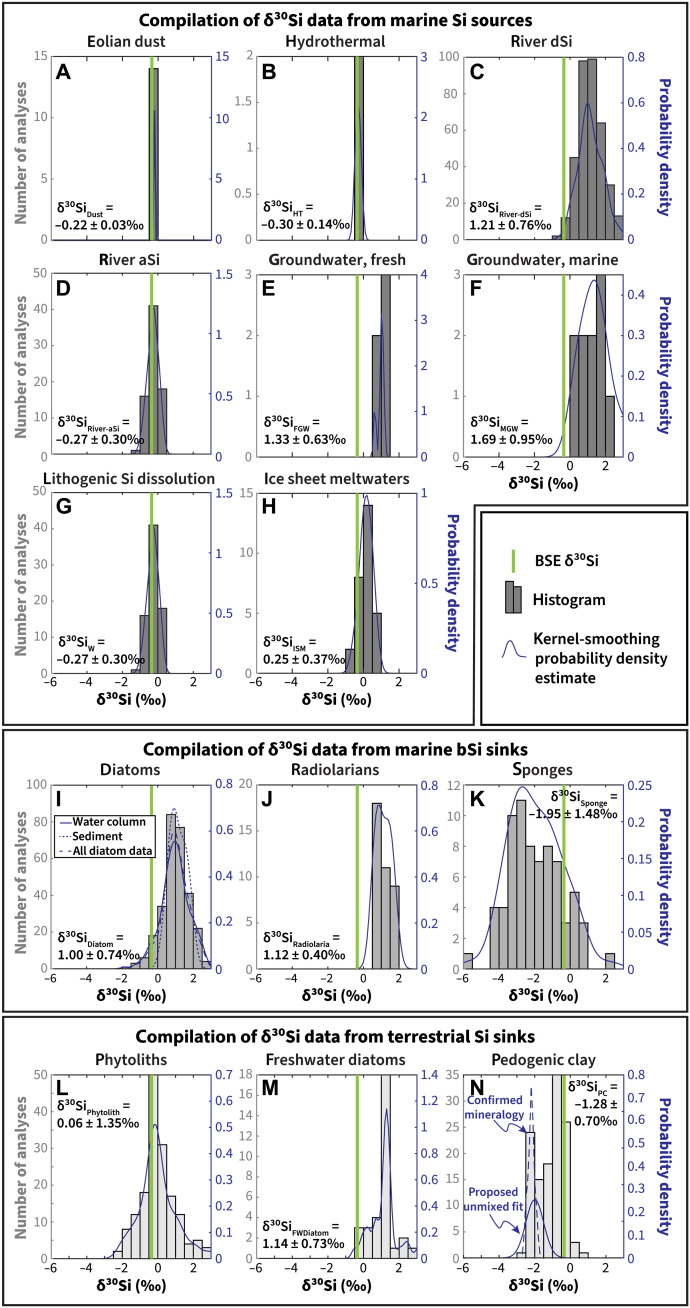
Distributions of δ^30^Si values from our compilation of marine Si sources, marine Si sinks, and terrestrial sinks corresponding to sources and sinks. In each plot, the left *y* axis and gray bars illustrate a histogram of the δ^30^Si data, and the right *y* axis and blue lines indicate probability density function estimates of each δ^30^Si distribution. The solid vertical line indicates the δ^30^Si value of BSE. (**A**) to (**H**) and (**I**) to (**K**) refer to marine Si sources and marine Si sinks, respectively, whereas (**L**) to (**N**) refer to terrestrial Si sinks.

To provide δ^30^Si constraints on the modern silica cycle, we compiled >3000 δ^30^Si measurements of modern materials from 74 studies (tableS1), enumerated as following: marine silica sinks: diatoms: *n* = 240 from water column samples and *n* = 50 from sediments; siliceous sponge spicules: *n* = 72; marine silica sources: aeolian dust: *n* = 14, assuming that this source is well-represented by loess; hydrothermal fluids: *n* = 2; river dSi: *n* = 372, including a subset of *n* = 23 samples from river mouths; river aSi: *n* = 75; submarine groundwater discharge (SGD): *n* = 31, including subsets of *n* = 11 marine SGD [salinity, >25 parts per thousand (ppt)] and *n* = 5 freshwater SGD (salinity, <4 ppt); and (sub)polar glacial meltwaters: *n* = 40; and terrestrial silica sinks: secondary clay minerals formed in soils (hereafter, “pedogenic clays”): *n* = 104; plant phytoliths: *n* = 216; and freshwater diatoms: *n* = 33. Because of lack of data, low temperature silicate weathering (a marine silica source) was assumed to have a similar δ^30^Si distribution as river aSi (i.e., aSi, fraction in river suspended sediment which dissolves rapidly over minutes to hours along a salinity gradient). Compiled δ^30^Si data are summarized in tableS1, and δ^30^Si distributions are shown in Fig. 1. Seawater dSi, marine porewater dSi, and radiolarian test δ^30^Si values are not used in the inverse model as they are not a source or sink of dSi to the global ocean (seawater or marine porewater) or are an unconstrained but likely very small sink (radiolarians) but have been compiled here and are available in the Supplementary Materials.

### Estimating δ^30^Si_RW_

Following the logic in a recent Precambrian silica cycling study (*38*), we designed a Monte Carlo inverse modeling approach to estimate an unknown (e.g., the δ^30^Si value of a source/sink or the size of a source/sink in terms of a flux) based on the distributions of δ^30^Si values of each of source and sink drawn from our data compilation and the size of each source and sink (*F*), including uncertainty. For most of our simulations, we used flux estimates from the most recent marine silica cycle summary (*12*), which discussed prior iterations, synthesized a number of recent publications that amended portions of the marine summary, and updated estimates of biogenic silica production. We applied this approach to test the underlying assumptions about Si isotope mass balance in the silica cycle. Specifically, because we lack direct constraints on the δ^30^Si value of reverse weathering products (δ^30^Si_RW_), we inverted the data to estimate δ^30^Si_RW_ assuming isotopic mass balance between marine Si sources and sinks. By evaluating whether these δ^30^Si_RW_ estimates are reasonable, we assess whether the underlying assumption of Si isotopic mass balance is correct.

**Fig. 2.**
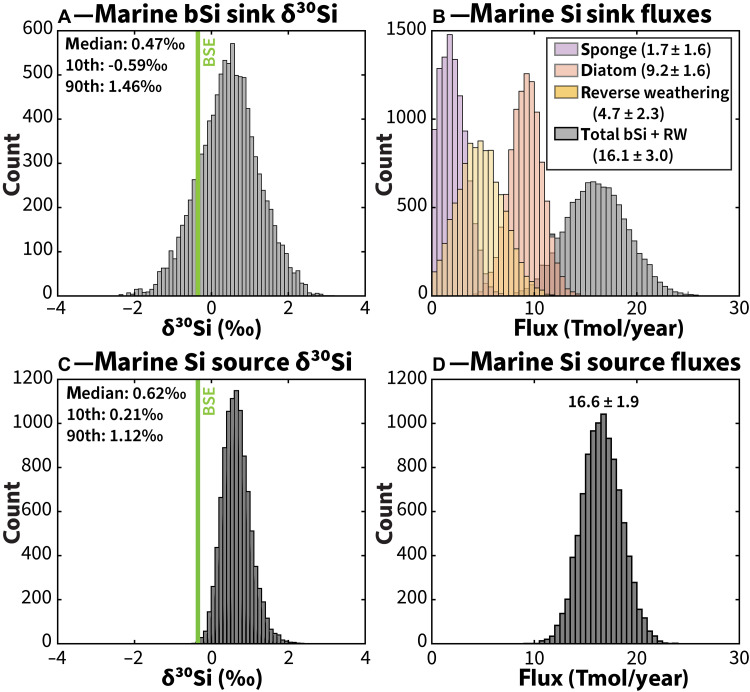
Histogram summary diagrams of marine silica sources and sinks with respect to δ^30^Si values and fluxes. Histograms show flux-weighted estimates of (**A**) marine bSi sink δ^30^Si value and flux estimates for (**B**) marine Si sinks using fluxes from the most recent marine silica budget (*12*) as well as flux-weighted estimates of (**C**) marine Si source δ^30^Si value and (**D**) marine Si sources using fluxes from the most recent marine silica budget (*12*). In (A) and (C), the vertical line indicates BSE δ^30^Si value. In (B), purple, orange, and yellow bars indicate estimates of sponge, diatom, and reverse weathering fluxes, respectively, while gray bars show the sum of these three sinks. Note that flux-weighted δ^30^Si estimates in (A) exclude reverse weathering due to a lack of constraints on the δ^30^Si value of this sink.

The distribution of flux-weighted averages of marine biogenic silica (bSi) sinks (median, 0.47‰; 10th percentile, −0.59‰; 90th percentile, 1.46‰) is weighted toward lower δ^30^Si values than the flux-weighted averages of marine Si sources (median, 0.62‰; 10th percentile, 0.21‰; 90th percentile, 1.12‰) (Fig. 2, A and C). Furthermore, both marine sources and sinks are offset to higher δ^30^Si values than BSE. It is evident from the δ^30^Si distributions that there must be another marine sink in addition to diatoms and sponges if the marine silica budget is in Si isotopic mass balance.

**Fig. 3.**
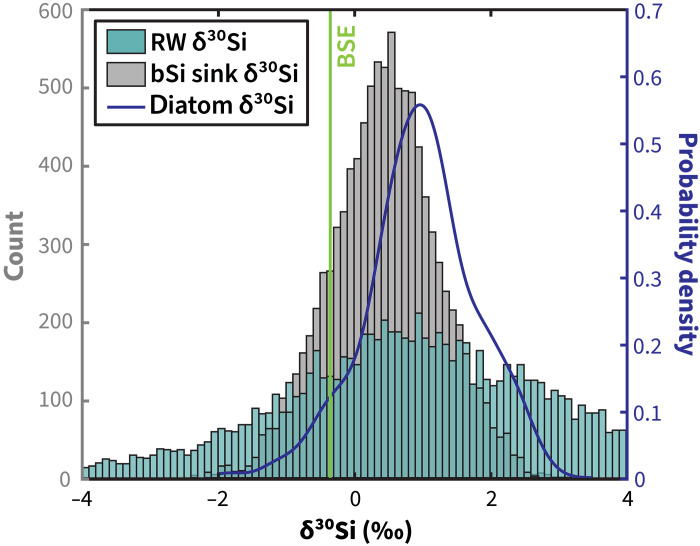
Histogram of inverse model estimates of flux-weighted δ^30^Si value of authigenic clays formed via reverse weathering. Flux-weighted δ^30^Si_RW_ estimates (teal bars, left *y* axis) have a similar mean as the distribution of δ^30^Si_diatom_ values in our compilation (blue line, right *y* axis) and are generally shifted to higher δ^30^Si values relative to flux-weighted marine bSi sink δ^30^Si values (gray bars, left *y* axis) and relative to δ^30^Si_BSE_ (vertical line). RW, reverse weathering.

Although it has been commonly assumed that δ^30^Si_RW_ would be offset to lower δ^30^Si values from the materials being altered (in this case, δ^30^Si_Diatom_) by 1 to 2‰ (*5*, *39*), our model results suggested that global average δ^30^Si_RW_ composition (mean, 1.31 ± 2.96‰) does not differ strongly from global average δ^30^Si_Diatom_ values (mean, 1.00 ± 0.74‰) (Fig. 3). This finding is robust to different model parameterizations and assumptions (figs. S1 and S2), including: (i) *F_RW_* (existing best estimate from the most recent marine silica budget (*12*) versus estimate requiring mass balance between source and sink fluxes), (ii) varying the size of the diatom sink (e.g., 7 or 14 Tmol/year burial), (iii) the representativeness of the diatom δ^30^Si data (e.g., assuming burial in different ocean basins or water depths was the most accurate representation of the global δ^30^Si_diatom_ sink), (iv) the representativeness and parameterization of river dSi δ^30^Si data, and (v) the representativeness of the sponge δ^30^Si data (e.g., assuming a substantially narrower range and higher mean δ^30^Si than in our compiled data) (see Materials and Methods).

### Terrestrial silica sinks

We also used our model to examine another poorly constrained sink—silica burial in terrestrial settings—by assuming that the combination of marine and terrestrial sinks must be in Si isotopic mass balance with BSE. Flux-normalized δ^30^Si values of both marine silica sources and sinks are not in isotopic mass balance with BSE (Fig. 2), although they are in isotopic mass balance with each other. This indicates that an additional silica sink is required for the global silica cycle to be in Si isotopic mass balance with BSE. We decided to assess whether terrestrial silica sinks were the most important and most likely “missing” sink: Some fraction of the silica released from silicate minerals via silicate weathering on continents is retained on continents in the form of secondary clay minerals in soils (hereafter referred to as “pedogenic clay,” *F*_pedogenic clay_), plant bSi (phytoliths, *F*_phytoliths_), and freshwater diatoms (*F*_freshwater diatoms_) (*40*). Using compiled δ^30^Si data from these three terrestrial Si sinks (Table 1), we estimated the size of the total terrestrial Si sink in terms of a flux magnitude (*F*_terr-sinks_). Rather than imposing assumptions on the absolute or relative sizes of each of the individual terrestrial sinks, we explored the parameter space of possible three-component mixtures.

**Fig. 4.**
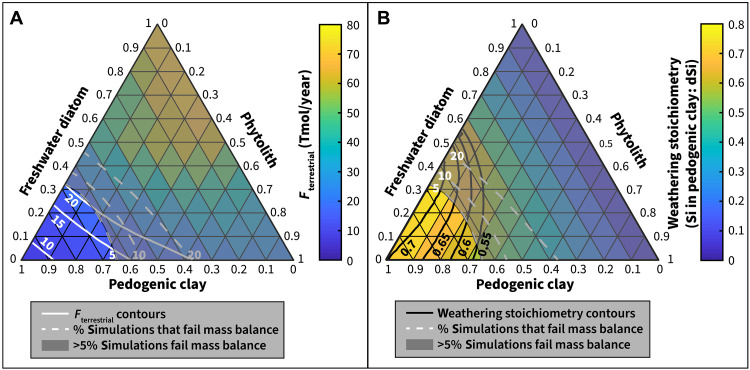
Ternary contour plots of the calculated magnitude of the terrestrial Si sink and silicate weathering stoichiometry. Ternary plots show the magnitude of the terrestrial Si sink (**A**) and silicate weathering stoichiometry (**B**) for all possible mixtures of the three terrestrial Si sink components (top corner: freshwater diatoms; bottom left corner: pedogenic clay; bottom right corner: phytoliths). Combinations for which >5% of simulations fail mass balance are grayed out; contours of 5, 10, and 20% of simulations failing mass balance are shown in dashed lines. In (A), contours of *F*_terr-sinks_ (in teramoles per year) are shown in white solid lines. In (B), contours of weathering stoichiometry are shown in black solid lines.

Compiled δ^30^Si distributions for each terrestrial Si sink are shown in Fig. 1 (L to N). Since the flux-weighted marine Si sink δ^30^Si distributions (both with and without reverse weathering products) are almost all greater than δ^30^Si_BSE_, the flux-weighted terrestrial Si sink δ^30^Si distribution must be less than δ^30^Si_BSE_ to fulfill the presumed condition that the combined sink is in Si isotopic mass balance with BSE. However, the distributions of δ^30^Si values of both freshwater diatoms and phytoliths are biased to values similar to or greater than δ^30^Si_BSE_ (Fig. 1, L and M). Therefore, many combinations of the three terrestrial sinks could not be isotopically mass balanced—primarily combinations that were composed mostly of phytoliths and freshwater diatoms, with a smaller contribution from pedogenic clays. We applied a conservative threshold and considered that only combinations for which <5% of the simulations failed mass balance were likely solutions. Given this constraint, our model results predicted that *F*_terr-sinks_ is ~8.5 to 20 Tmol/year (median, 13.6 Tmol/year; 10th percentile, 10.2 Tmol/year; 90th percentile, 18.3 Tmol/year) (Fig. 4A).

We unmixed the *F*_terr-sinks_ estimates for each of the possible three-component mixtures to yield estimates for the sizes of pedogenic clay (*F*_pedogenic clay_ = 8.6 to 13.9 Tmol/year; median, 10.1 Tmol/year; 10th percentile, 9.0 Tmol/year; 90th percentile, 12.4 Tmol/year), phytolith (*F*_phytolith_ = ~0 to 4.7 Tmol/year; median, 1.5 Tmol/year; 10th percentile, 0.2 Tmol/year; 90th percentile, 3.6 Tmol/year), and freshwater diatom (*F*_freshwater diatom_ = ~0 to 6.6 Tmol/year; median, 1.4 Tmol/year; 10th percentile, 0.2 Tmol/year; 90th percentile, 4.3 Tmol/year) sinks (fig. S3).

We used the estimates of *F*_pedogenic clay_ to solve for the average stoichiometry of weathering reactions that produced those pedogenic clay minerals for each of the mixtures. Our model demonstrated that the Si stoichiometry of silicate mineral weathering reactions required for the global silica sinks to be in isotopic mass balance with BSE is 0.54 to 0.74 moles of Si stored in pedogenic clays per mole of silica released as dSi (median, 0.66; 10th percentile, 0.59; 90th percentile, 0.72) (Fig. 4B), consistent with reactions such as the incongruent weathering of K-feldspar, producing kaolinite (clay storage ratio = Si in clay: dSi = 0.5; Eq. 4) or the incongruent weathering of plagioclase, producing halloysite (e.g., clay storage ratio = 0.75; Eq. 5) (*17*). Both these reactions are commonly used in global carbon budgets of silicate weathering, but our δ^30^Si mass balance approach provides an independent method to assess whether these reactions are consistent with silica cycle fluxes. The size of *F*_pedogenic clay_ and stoichiometry of storage versus release implies that 12.3 to 27.5 Tmol/year of Si (median, 19.1 Tmol/year; 10th percentile, 14.4 Tmol/year; 90th percentile, 24.5 Tmol/year) is freed as dSi via terrestrial silicate mineral weathering (*F*_terr_weathering_; does not include processes associated with saline water), much higher than what has been used in the past to model the modern silica cycle [e.g., (*40*)]. Another way of estimating *F*_terr_weathering_ is to consider that if ~70% of initial dSi released through weathering is trapped in terrestrial secondary silicate minerals and net dSi flux (remaining 30%) to ocean is 6.2 Tmol/year, then gross dSi produced during silicate mineral weathering before formation of secondary minerals should be ~21 Tmol/year. These values for dSi release via silicate mineral weathering, 19.1 Tmol/year and 21 Tmol/year, are in close agreement.

## DISCUSSION

### Evaluating Si isotopic mass balance of marine silica budgets

**Fig. 5.**
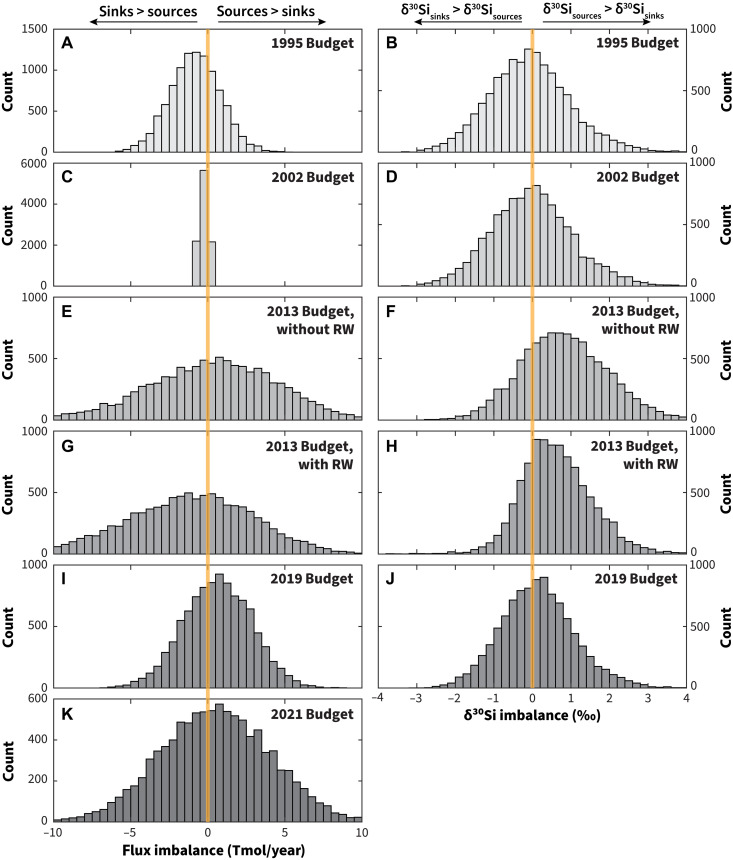
Histograms of imbalances in marine fluxes and marine flux-weighted δ^30^Si values for older marine silica budgets. Histograms of fluxes (left column) (**A**, **C**, **E**, **G**, **I**, and **K**) and flux-weighted δ^30^Si values (right column) (**B**, **D**, **F**, **H**, and **J**) compare estimates based on published budgets from 1995 (*41*), 2002 (*65*), 2013 (*34*), 2019 (*66*), and 2021 (*12*). Orange vertical lines in each panel highlight the conditions where the fluxes and flux-weighted δ^30^Si values are balanced. Calculations for the 2013 budget (*34*) (E to H) are compared excluding and including reverse weathering (“without RW” or “with RW,” respectively) because the calculation including reverse weathering requires using the estimate of δ^30^Si_RW_ from our model results.

Summaries of the marine silica cycle has evolved over the past 30 to 40 years, and some of the major representations are summarized in Table 1. These various fluxes to and from the ocean, or “budgets,” were tested for flux and isotope mass balance with our model. Our results (Fig. 5) indicate that iterations such as the ~20-year-old summaries (*41*, *42*) appear balanced with respect to Si isotopes. However, these models did not include sponge spicules as a sink and are therefore likely only balanced because they are incomplete. The 2013 marine silica cycle summary (*34*) offered some major changes to the reactive silica cycle summary in the form of additional sources aSi, higher flux from hydrothermal systems, seafloor weathering, lithogenic dissolution, and fresh submarine groundwater discharge. Another major revision was the addition of two marine silica sinks that had not been included in previous budgets: siliceous sponge spicules (>30% of the global budget) and reverse weathering products (~15% of the global budget). With respect to sponges (median, −2.14‰; 10th percentile, −3.69‰; 90th percentile, 0.09‰), a large sponge burial flux of a magnitude to balance the summary will create an imbalance in the silicon isotope mass balance by driving the flux-weighted silica sink δ^30^Si value lower relative to the flux-weighted silica source δ^30^Si values (Fig. 5). This is apparent in the distributions of flux-weighted δ^30^Si imbalance of simulations using the 2013 budget (Fig. 5, F and H) (*34*), which, unlike other budgets, are not centered on zero, reflecting that the increased magnitude of the sponge sink in that budget resulted in more simulations where δ^30^Si_sources_ > δ^30^Si_sinks_.

The model simulations indicate that the distribution of the median δ^30^Si_RW_ value (median, 1.06‰) needed to balance the budget closely matches the median δ^30^Si_Diatom_ value (median, 1.00‰), although the distribution of δ^30^Si_RW_ estimates (10th percentile, −1.77‰; 90th percentile, 4.8‰) encompasses a greater range than δ^30^Si_diatom_ (10th percentile, 0.13‰; 90th percentile, 1.95‰). Model solutions at the lower end of the distributions in the δ^30^Si_RW_ values would be consistent with some dSi released via sponge or aSi (tableS1) dissolution being sequestered into reverse weathering products. Model solutions at the higher end of the distributions could result from an imbalance in the most recent 2021 summary (*12*). However, this model result (that median δ^30^Si_RW_ is similar to median δ^30^Si_Diatom_) is the same if the fluxes are forced to be in mass balance (fig. S1B). Some findings of reverse weathering along the shelf (*5*, *42*, *43*), the open ocean (*44*), and Hadal zone (*45*) were not quantified. Efflux of high δ^30^Si values to the water column via alteration of serpentine to an authigenic clay by seawater (*10*) and deep submarine groundwater inputs (*46*) point to additional sink and source terms, respectively, that were also not quantitatively considered in the 2021 summary (*12*). These additional terms may explain the higher end of the model estimates (Fig. 3). Our model finding about δ^30^Si_RW_ is most easily explained by interpreting that almost all the dSi that is released via diatom frustule dissolution is consumed via reverse weathering reactions. If this can be confirmed with more data, implicit in this finding is that reverse weathering reaction rates occur on time scales that are less than the residence time of dSi in the global ocean (i.e., <7.7 ka), consistent with direct observations of authigenic clay formation rates (*22*, *23*) and the activity of cosmogenic ^32^Si in sedimentary reactive Si pools (*24*, *25*). Also implicit in this finding is the possibility that the δ^30^Si_RW_ distribution may change on glacial-interglacial time scales as (i) the fraction of δ^30^Si_Rivers_ that is sourced from proglacial rivers varies and the relative contribution from δ^30^Si_ISM_ increases or decreases through time; (ii) the δ^30^Si_Diatom_ distribution changes over glacial-interglacial time scales (*47*); and (iii) delivery of reactants needed to form reverse weathering products become uncoupled with sea level rise and retreat (*28*).

### Evaluating model estimates of Si storage in pedogenic clays with Li isotopes

The predicted fraction of dSi that is released on land and forms secondary minerals (Fig. 4) can be tested using the stable Li isotope record. We can use the riverine Li record to test whether the modeled relative fraction of secondary mineral formation needed to balance the δ^30^Si composition of river water is reasonable. Lithium is a silicate mineral weathering product, whose major sinks in the terrestrial hydrologic cycle are via incorporation into secondary silicate mineral phases (*48*) and Fe oxides (*49*, *50*). The riverine record of lithium isotopes can be used to provide constraints on silicate mineral weathering rates at a global scale (*51*–*53*). We use a simple Rayleigh fractionation model to the global riverine dissolved Li inventory and composition, using fractionation factors derived for secondary mineral formation. This model can be applied by making the simplifying assumption that there is no additional supply of Li by mineral dissolution and there is net removal by uptake into secondary mineral phases

$$\delta^{7}{\mathrm{Li}}_{\mathrm{river}s}=\delta^{7}{\mathrm{Li}}_{0}+1000(\alpha -1)\ln(f_{\mathrm{river}}^{\mathrm{Li}})$$ (6)

In Eq. 6, δ^7^Li_rivers_ is the global average river end-member dissolved Li isotopic composition entering the ocean [i.e., 23‰ (*50*, *54*)], δ^7^Li_0_ is the initial δ^7^Li value of Li upon dissolution from a primary mineral [i.e., 0‰ (*50*)], α is the fractionation factor between $\frac{{}^{7}\mathrm{Li}}{{}^{6}\mathrm{Li}}$ ratios as Li is precipitated from river water, and $f_{\mathrm{river}}^{\mathrm{Li}}$ is the fraction of dissolved Li remaining in river water. Here, we use an α value range of 0.983 to 0.990, which are average experimentally determined Li isotope fraction factors during the formation of clays that is consistent between studies (*55*–*57*). Applying Eq. 6 to derive the fraction of dissolved Li remaining in river water returns an $f_{\mathrm{river}}^{\mathrm{Li}}$ value of 0.10 to 0.26. In other words, of the dissolved Li that is globally released during mineral weathering, ~75 to 90% is removed into a secondary mineral phase. This value is consistent with our estimate that ~70% of dSi released through primary silicate mineral weathering forms secondary minerals.

### Carbon cycle modeling implications and applications

Chemical weathering of silicate minerals, CO_2_ drawdown, and impacts to climate are modeled with assumed weathering congruencies (i.e., an assumed dSi production to CO_2_ consumption ratio). The stoichiometry of dSi:CO_2_ (e.g., Eqs. 4 and 5) varies in modeling efforts depending on the mineral phase that is chosen to represent silicate mineral weathering (*17*, *30*). Consider that during incongruent weathering of K-feldspar to kaolinite (Eq. 4), the Si:CO_2_ molar ratio of consumption is 2:1, i.e., for every 10 Tmol/year of dSi released during weathering, approximately 5 Tmol/year HCO_3_^−^ is produced or CO_2_ consumed. During congruent weathering, the Si:CO_2_ molar ratio of consumption is 1:2 (Eq. 1), i.e., for every 5 Tmol/year of dSi released during silicate mineral weathering, 10 Tmol/year HCO_3_^−^ is produced or CO_2_ consumed. We can compare our estimates of dSi release (i.e., ~19 to 21 Tmol/year) to global alkalinity budgets and the portion of alkalinity that is estimated to originate from silicate mineral weathering. The global alkalinity river flux from weathering of carbonate rocks is ~17.3 Tmol/year of 30.1 Tmol/year total (*58*). For all other lithologies, the fluxes are 1.1 Tmol/year (sands and sandstones), 8.6 Tmol/year (shales), 1.5 Tmol/year (shield), 0.2 Tmol/year (acid volcanic rocks), and 1.5 Tmol/year (basalts), which sum to 12.9 Tmol/year of HCO_3_^−^ that is released from noncarbonate rock weathering. The global Si:CO_2_ molar ratio of these two fluxes is ~1.5 to 1.6, which is closer to the ratio predicted from incongruent weathering reactions on a global scale. This is the stoichiometric Si:CO_2_ ratio we recommend for modeling chemical silicate mineral weathering on a global scale for modern Earth.

**Fig. 6.**
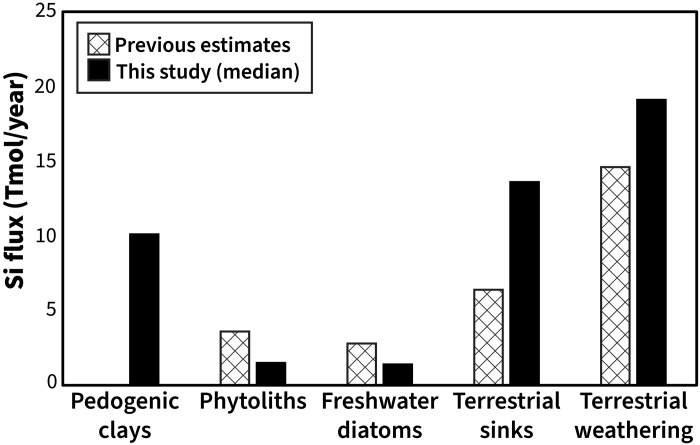
Model estimates of individual fluxes of terrestrial reactive pedogenic clay, phytolith, freshwater diatom, and total terrestrial sinks. Estimates from this study compared to previous estimates (*40*, *59*). A comparison of our model estimate of total terrestrial weathering is also included.

It should also be noted that the median estimates for *F*_phytolith_ and *F*_freshwater diatom_ are ~50% lower than previous estimates from box modeling—3.6 and 2.8 Tmol/year, respectively (*40*). On the basis of available data, our model results suggest that <10% of the total silicate weathering dSi flux comprises the terrestrial phytolith sink, instead of ~25% (*40*, *59*). Freshwater diatom burial sink is <10% of the total silicate weathering dSi flux as well (Table 2). Instead, burial in the form of pedogenic clays far outweighs the magnitude of the other two sinks (Fig. 6).

**Table 2.** Model estimates of individual terrestrial reactive Si sinks (*F*_pedogenic clay_, *F*_phytolith_, and *F*_freshwater diatom_), total terrestrial reactive Si sink (*F*_terr-sinks_), and total initial Si weathering flux (*F*_terr weathering_).

Reactive Si pool	Flux (Tmol/year)	Median (Tmol/year)	10th percentile (Tmol/year)	90th percentile (Tmol/year)
*F*_pedogenic clay_	8.6–13.9	10.1	9.0	12.4
*F*_phytolith_	~0–4.7	1.5	0.2	3.6
*F*_freshwater diatom_	~0–6.6	1.4	0.2	4.3
F_terr-sinks_	8.5–20	13.6	10.2	18.3
*F*_terr weathering_	12.3–27.5	19.1	14.4	24.5

### Implications for deep time applications of δ^30^Si analyses

Our findings have substantial implications for applications of δ^30^Si analyses to reconstruct the silica cycle in deep time. For example, a recent Precambrian silica cycling study (*38*) proposed the application of inverse modeling to constrain the sizes of marine silica sinks using distributions of δ^30^Si measurements from various sinks. This approach relies on the assumption of Si isotopic mass balance of the global silica cycle with BSE, which our results support. Specifically, our model results support the interpretation that the sources and sinks in the marine silica cycle are in Si isotopic mass balance with each other. Furthermore, the consistency of our estimates of silicate weathering fluxes with other independent estimates (see the previous section) supports the interpretation that the global silica cycle is in isotopic mass balance with BSE, because our estimates are conditioned on that assumption.

However, our findings also suggest that authigenic clays formed via reverse weathering reactions of precursor biogenic opal (i.e., diatom frustules) might not differ significantly in their δ^30^Si distributions from those precursors. On the one hand, this suggests that, in lieu of direct measurement methods, δ^30^Si values of authigenic clays formed via reverse weathering could be estimated from δ^30^Si distributions of diatomaceous sediments. However, on the other hand, the sizes of two sinks with indistinguishable δ^30^Si distributions cannot be differentiated—the inverse modeling approach could only be used to reconstruct the sum of the two sinks but not the relative contributions of each to that sum. Would the same process dominate before the evolution of diatoms? Diatom frustules are more reactive than radiolarian tests or sponge spicules (*37*, *60*), but in the absence of diatoms, would these other siliceous skeletal components also have transformed quantitatively to authigenic clays? Or would these reactions have produced clay minerals with distinct δ^30^Si compositions? More work is needed to answer these questions. Specifically, more observations of δ^30^Si values of reverse weathering products from modern sediments, particularly in locations where there is near total conversion of diatoms to authigenic clays are needed to confirm our model results. Experiment and field observations of alteration of siliceous sponge spicules and radiolarian tests are also needed to understand how to interpret Mesozoic and Paleozoic δ^30^Si records with respect to reconstructing fluxes via inversion modeling. Until the answers to these questions are known, we suggest that it be assumed that authigenic clays formed via reverse weathering reactions may not have had distinct δ^30^Si compositions from the siliceous skeletal components from which they formed across the Phanerozoic geological record.

Our findings also inform how inverse modeling of the marine silica cycle via δ^30^Si compositions should be applied and interpreted for the Precambrian rock record. Assuming that, along with cherts and iron formations, reverse weathering products were a major silica sink in Precambrian oceans (*29*, *30*, *38*), the inverse modeling approach requires that the δ^30^Si distributions of each of these three silica sinks be distinct to reconstruct their relative fluxes. Since there are no direct measurements of the δ^30^Si compositions of reverse weathering products from the rock record, a recent Precambrian silica cycling study (*38*) assumed that these authigenic clays might have δ^30^Si distributions offset from those of contemporaneous chert by −2‰, by analogy to the relatively large Si isotopic fractionation factors associated with formation of authigenic clays in pedogenic environments. However, our results suggest that understanding the pathway of reverse weathering clay formation is critical. If reverse weathering products precipitated directly from seawater, as envisioned by some authors (*29*), then the assumption of a larger δ^30^Si offset from seawater δ^30^Si than that captured by chert may still be reasonable. However, if some (or all) of these authigenic clays formed via alteration of an abiotic aSi precursor (i.e., a shared precursor to chert and/or banded iron formation [BIF]), then we must question the assumption that reverse weathering products are isotopically distinct from chert and/or BIF. A better understanding of the mechanisms of marine clay authigenesis in Precambrian seawater and methods to uniquely identify and measure the δ^30^Si compositions of reverse weathering products are both needed to advance our ability to interpret the Precambrian δ^30^Si record.

## MATERIALS AND METHODS

### Mass balance model

For the marine silica cycle, Si isotope mass balance can be expressed mathematically as in Eq. 7 below.

The left-hand side terms are the silica sources: aeolian dust, hydrothermal fluids (HT), dSi in rivers, aSi in rivers, fresh and marine submarine groundwater discharge (FGW and MGW), dissolution of lithogenic silica (W), and silica from glacial sources (ISM). The right-hand side terms are the silica sinks: bSi in the form of diatoms and sponge spicules and authigenic clays formed through reverse weathering reactions (RW).

To invert the data to estimate δ^30^Si_RW_, we compared two approaches. In the first approach, we solved Eq. 7 for a single unknown, δ^30^Si_RW_ as in Eq. 8 below.

$$\frac{F_{\mathrm{eolian}}\delta^{30}{\mathrm{Si}}_{\mathrm{eolian}}+F_{\mathrm{HT}}\delta^{30}{Si}_{\mathrm{HT}}+F_{\mathrm{river}\;\mathrm{dSi}}\delta^{30}{Si}_{\mathrm{river}\;\mathrm{dSi}}+F_{\mathrm{river}\;\mathrm{aSi}}\delta^{30}{Si}_{\mathrm{river}\;\mathrm{aSi}}+F_{\mathrm{FGW}}\delta^{30}{\mathrm{Si}}_{\mathrm{FGW}}+F_{\mathrm{MGW}}\delta^{30}{\mathrm{Si}}_{\mathrm{MGW}}+F_{W}\delta^{30}{\mathrm{Si}}_{W}+F_{\mathrm{ISM}}\delta^{30}{\mathrm{Si}}_{\mathrm{ISM}}}{F_{\mathrm{eolian}}+F_{HT}+F_{river\;dSi}+F_{river\;aSi}+F_{FGW}+F_{MGW}+F_{W}+F_{ISM}}=\frac{F_{\mathrm{diatom}}\delta^{30}{\mathrm{Si}}_{\mathrm{diatom}}+F_{\mathrm{sponge}}\delta^{30}{\mathrm{Si}}_{\mathrm{sponge}}+F_{\mathrm{RW}}\delta^{30}{\mathrm{Si}}_{\mathrm{RW}}}{F_{\mathrm{diatom}}+F_{\mathrm{sponge}}+F_{\mathrm{RW}}}$$ (7)

$$\begin{array}{rl} & \delta^{30}{Si}_{\mathrm{RW}}=\frac{1}{F_{\mathrm{RW}}}∙[(F_{\mathrm{diatom}}+F_{\mathrm{sponge}}+F_{\mathrm{RW}})∙\\ & \frac{F_{\mathrm{eolian}}\delta^{30}{Si}_{\mathrm{eolian}}+F_{\mathrm{HT}}\delta^{30}{Si}_{\mathrm{HT}}+F_{\mathrm{river}\;\mathrm{dSi}}\delta^{30}{Si}_{\mathrm{river}\;\mathrm{dSi}}+F_{\mathrm{river}\;\mathrm{aSi}}\delta^{30}{Si}_{\mathrm{river}\;\mathrm{aSi}}+F_{\mathrm{FGW}}\delta^{30}{Si}_{\mathrm{FGW}}+F_{\mathrm{MGW}}\delta^{30}{Si}_{\mathrm{MGW}}+F_{W}\delta^{30}{Si}_{W}+F_{\mathrm{ISM}}\delta^{30}{Si}_{\mathrm{ISM}}}{F_{eolian}+F_{HT}+F_{river\;dSi}+F_{river\;aSi}+F_{FGW}+F_{MGW}+F_{W}+F_{ISM}}\\ & -F_{\mathrm{diatom}}\delta^{30}{Si}_{\mathrm{diatom}}+F_{\mathrm{sponge}}\delta^{30}{Si}_{\mathrm{sponge}}] \end{array}$$ (8)

In this approach, *F*_RW_ was constrained using the most recent marine silica summary estimate (*12*). Since that study (*12*) budget is not in perfect mass balance with respect to silica fluxes and because the Monte Carlo approach incorporates the uncertainty in each flux, this approach essentially forced the marine silica cycle to be in Si isotopic mass balance even when it was not in mass balance with respect to fluxes. Therefore, we designed a second approach to estimate δ^30^Si_RW_ that solves for *F*_RW_ first, by assuming mass balance of silica fluxes

$$F_{RW}=F_{eolian}+F_{HT}+F_{river\;dSi}+F_{river\;aSi}+F_{FGW}+F_{MGW}+F_{W}+F_{ISM}-(F_{diatom}+F_{sponge})$$ (9)

This *F*_RW_ estimate was then substituted into Eq. 7 to solve for δ^30^Si_RW_ and the resulting δ^30^Si_RW_ estimates compared with those from the first approach.

### Model sensitivity tests

Using this basic approach, we explored the sensitivity of δ^30^Si_RW_ estimates to key model assumptions, including (i) the size of the diatom sink, (ii) the representativeness of the diatom δ^30^Si data, (iii) the representativeness of river dSi δ^30^Si data, and (iv) the representativeness of the sponge δ^30^Si data. (i) With respect to the size of the diatom sink, we compared model δ^30^Si_RW_ estimates for smaller and larger *F*_Diatom_ (7 and 14 Tmol/year) (fig. S1, C and D); under both scenarios, we used Eq. 8 to determine *F*_RW_ assuming flux mass balance. (ii) With respect to addressing the representativeness of the diatom data, which is primarily from Southern Ocean samples, we explored whether surface seawater dSi δ^30^Si, for which there is better geographic data coverage, could be used to estimate diatom δ^30^Si values via the well-characterized diatom Si isotope fractionation factor. We explored the relationship between surface seawater dSi δ^30^Si and diatom δ^30^Si values and determined that, even in the Southern Ocean, using seawater dSi δ^30^Si to estimate diatom δ^30^Si values introduces more uncertainty due to Rayleigh-type fractionation (fig. S4). (iii) With respect to addressing possible bias in river dSi δ^30^Si due to changes in δ^30^Si along the length of each river (*61*), we compared model δ^30^Si_RW_ estimates using only dSi δ^30^Si data from river mouths (which we determined could more accurately reflect the signal of dSi δ^30^Si delivered to seawater, at the cost of reduced sample size) (fig. S1E) and developing a flux-weighted dSi δ^30^Si estimate by matching each river in our database to catchments (COastal Segmentation and related CATchments) following a coastal segmentation study (*62*) and weighting each catchment by dSi flux following a spatial silica flux study (figs. S1F and S5) (*62*, *63*). (iv) With respect to addressing the representativeness of the sponge δ^30^Si data, we explored how the model δ^30^Si_RW_ estimates were affected by adopting δ^30^Si_sponge_ distributions with significantly narrower ranges and higher mean δ^30^Si values than the data in our compilation. These alternative parameterizations of δ^30^Si_sponge_ reflect an assumption that the sponge δ^30^Si data in our compilation is biased toward low δ^30^Si values due to an oversampling effect of sponges living in waters with higher dSi concentration and/or lower dSi δ^30^Si values. We used two alternative δ^30^Si_sponge_ parameterizations assuming normal distributions with mean values of either −1 or 0.5‰ and SD of 0.5‰, both of which have some overlap with but are a poor fit to our compilation δ^30^Si_sponge_ data.

### Modeling terrestrial silica sinks

The δ^30^Si distribution of clays from terrestrial sediments in our compilation is bimodal, with one of the peaks closely aligned with the δ^30^Si value of BSE. Since a number of the studies included in this compilation defined “clay” as a size fraction rather than by mineralogy, we considered it was likely that the peak in δ^30^Si_PC_ values near δ^30^Si_BSE_ reflected clay-sized primary silicate minerals (e.g., biotite, clay-sized quartz and feldspar grains, etc.) rather than pedogenic clay minerals, particularly given the reported ~−2‰ fractionation (*64*) associated with formation of kaolinite. Therefore, we compared terrestrial sink size estimates assuming the full distribution of δ^30^Si_PC_ values from our compilation and a normal distribution of δ^30^Si_PC_ values with mean −2‰ and SD of 0.5‰, which matches the peak in the bimodal δ^30^Si_PC_ distribution that does not coincide with δ^30^Si_BSE_.

These terrestrial silica sink estimates were calculated by assuming that the sum of terrestrial and marine silica sinks is in isotopic mass balance with BSE

$$\delta^{30}{\mathrm{Si}}_{\mathrm{BSE}}=\frac{\delta^{30}{\mathrm{Si}}_{\mathrm{marine}}F_{\mathrm{marine}}+\delta^{30}{\mathrm{Si}}_{\mathrm{terr}-\mathrm{sinks}}F_{\mathrm{terr}-\mathrm{sinks}}}{F_{\mathrm{marine}}+F_{\mathrm{terr}-\mathrm{sinks}}}$$ (10)

where δ^30^Si_marine_ and δ^30^Si_terrestrial_ are flux-weighted averages of marine and terrestrial silica sinks, respectively. Eq. 10 can then be rearranged to solve for *F*_terrestrial_

$$F_{\mathrm{terr}-\mathrm{sinks}}=F_{\mathrm{marine}}\cdot \frac{\delta^{30}{\mathrm{Si}}_{\mathrm{marine}}-\delta^{30}{\mathrm{Si}}_{\mathrm{BSE}}}{\delta^{30}{\mathrm{Si}}_{\mathrm{BSE}}-\delta^{30}{\mathrm{Si}}_{\mathrm{terr}-\mathrm{sinks}}}$$ (11)

To estimate the global stoichiometry of silicate weathering reactions, we calculated the ratio of the flux of Si into storage as secondary clay minerals (*F*_pedogenic clay_) to the total flux of dissolved Si produced by silicate weathering, for which we used the sum of the fluxes of fluvial dSi + aSi, submarine groundwater discharge, ice sheet meltwaters, freshwater diatoms, and phytoliths.

## Supplementary Materials

### This PDF file includes:

Figs. S1 toS5 Table S1 References
