# Supplementary material for: Probing surface Earth reactive silica cycling using stable Si isotopes: Mass balance, fluxes, and deep time implications
Source: Sci Adv. 2023 Dec 6;9(49):eadi2440. doi: 10.1126/sciadv.adi2440 (PMC10699779; doi:10.1126/sciadv.adi2440)
Supplement: Supplementary file 1 — Figs. S1 toS5 Table S1 References [file sciadv.adi2440_sm.pdf]

Science Advances
AAAS

Supplementary Materials for

Probing surface Earth reactive silica cycling using stable Si isotopes: Mass balance, fluxes, and deep time implications

Shaily Rahman and Elizabeth J. Trower

Corresponding author: Shaily Rahman, shaily.rahman@colorado.edu

Sci. Adv. **9**, eadi2440 (2023)
DOI: 10.1126/sciadv.adi2440

This PDF file includes:

Figs. S1 to S5
Table S1
References

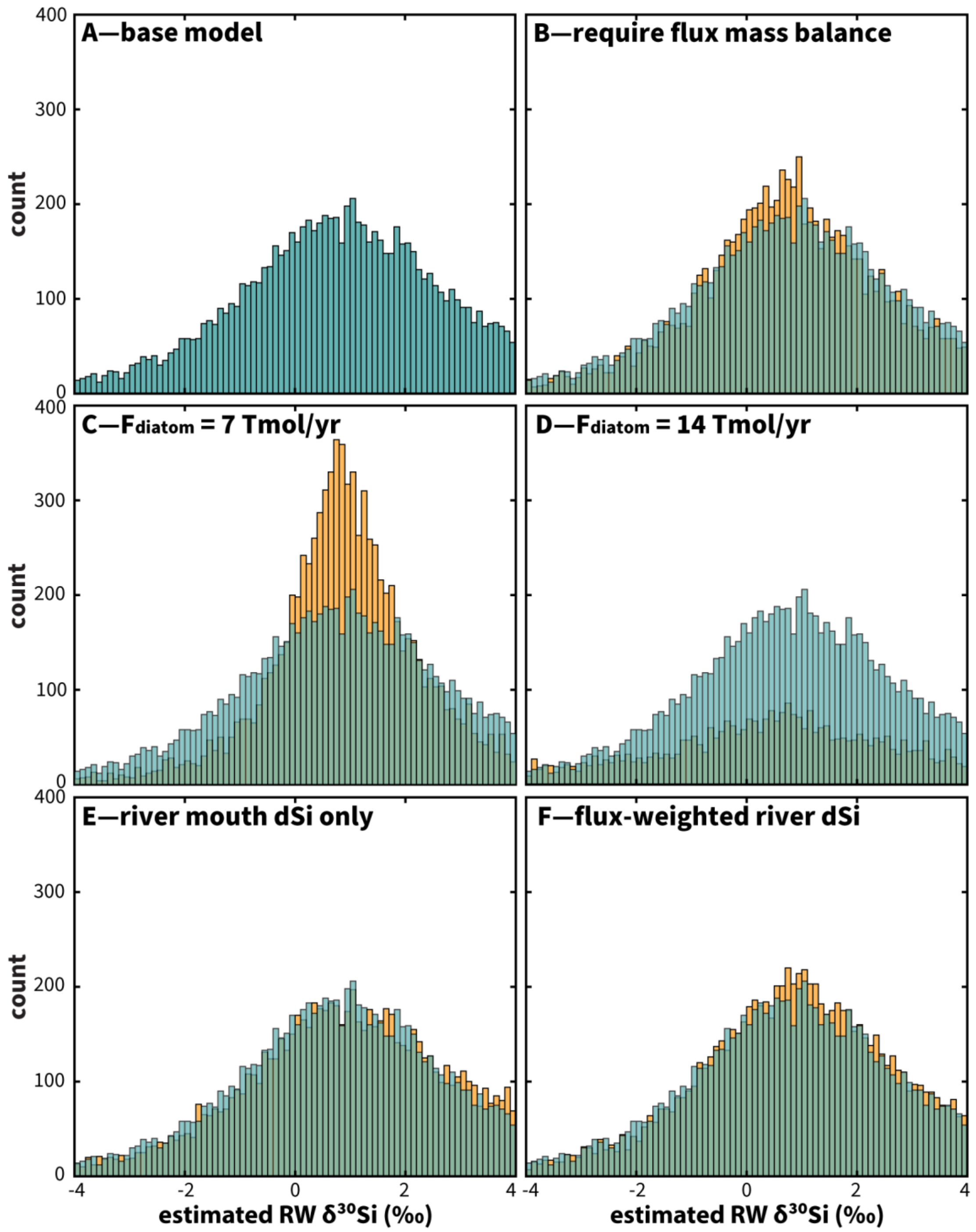

Fig. S1.

Sensitivity tests of inverse model estimates of flux-weighted $\delta^{30}Si_{RW}$ value. Each panel compares histogram of base model estimates presented and discussed in main text (A, teal bars in all panels) with histogram of model $\delta^{30}Si_{RW}$ estimates with different model parameterizations and/or assumptions: (B) model parameterization that requires marine sources and sinks be in flux balance (F_{RW} is calculated as the flux needed to balance the sinks); (C) smaller diatom sink than assumed in the base model; (D) larger diatom sink than assumed in the base model; (E) model

parameterization using only river mouth dSi $\delta^{30}Si$ data, which addresses possible bias in river dSi $\delta^{30}Si$ data caused by changes in dSi $\delta^{30}Si$ along the length of each river; and (F) model parameterization using a flux-weighted river dSi $\delta^{30}Si$ estimate (see Materials and Methods). Each histogram reflects the 10,000 simulations that were run for each model parameterization, although the x-axis has been cropped for clarity to only show a likely range of $\delta^{30}Si$ values.

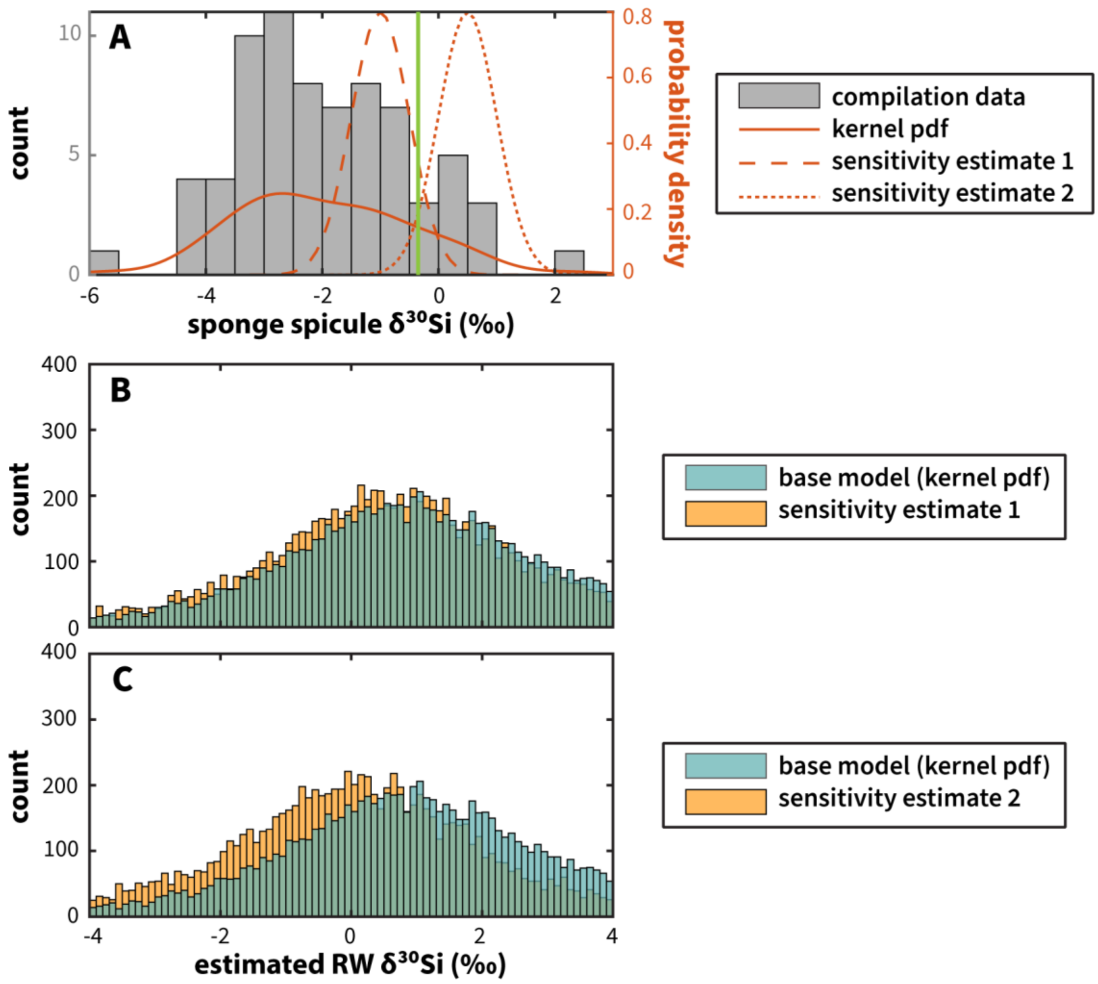

Fig. S2.

Sensitivity tests of inverse model estimates of flux-weighted $\delta^{30}Si_{RW}$ value to representativeness of the sponge $\delta^{30}Si$ data in our compilation. (A) Comparison of compilation data (gray histogram) and kernel-smoothing probability density function estimate (solid orange line) used in base model with two normal distributions used to test the effect of a sponge sink with a heavier mean value and narrower range of underlying data (dashed orange lines). (B) Comparison of base model output (teal histogram of predicted $\delta^{30}Si_{RW}$ values) with model output assuming sensitivity estimate 1 (mean -1‰, standard deviation 0.5‰), illustrating that the key result is not substantially similar. Note that this synthetic sponge $\delta^{30}Si$ estimate overlaps with a subpeak of the underlying data. (C) Comparison of base model output (teal histogram of predicted $\delta^{30}Si_{RW}$ values) with model output assuming sensitivity estimate 2 (mean 0.5‰, standard deviation 0.5‰). In this example, $\delta^{30}Si_{RW}$ is shifted to a range of lower values; however, this synthetic sponge $\delta^{30}Si$ estimate is both extreme and unrealistic. Without a clear mechanistic explanation of why the sponge silica sink might be offset to the extreme end of the range of underlying observations, we interpret that bias in the sponge sink is unlikely to significantly affect our key finding that the distribution of $\delta^{30}Si_{RW}$ must broadly match that of $\delta^{30}Si_{diatom}$.

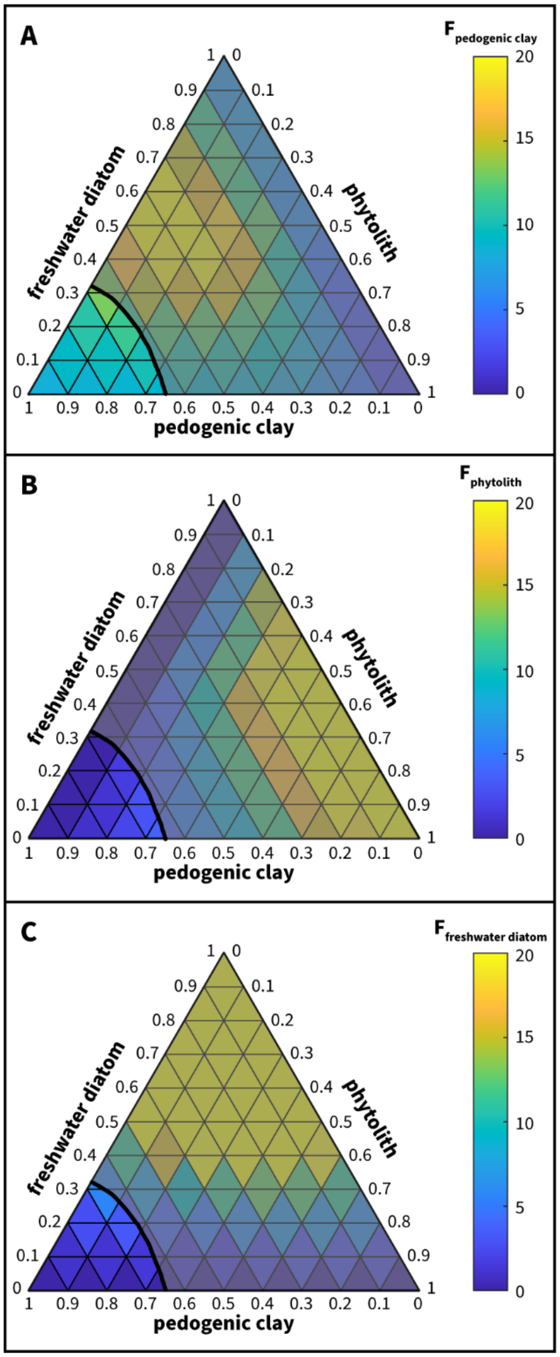

Fig. S3.

Ternary contour plots of calculated sizes of terrestrial Si sinks: (A) pedogenic clays, (B) phytoliths, and (C) freshwater diatoms. Grayed out areas correspond to mixtures for which >5% of simulations fail mass balance.

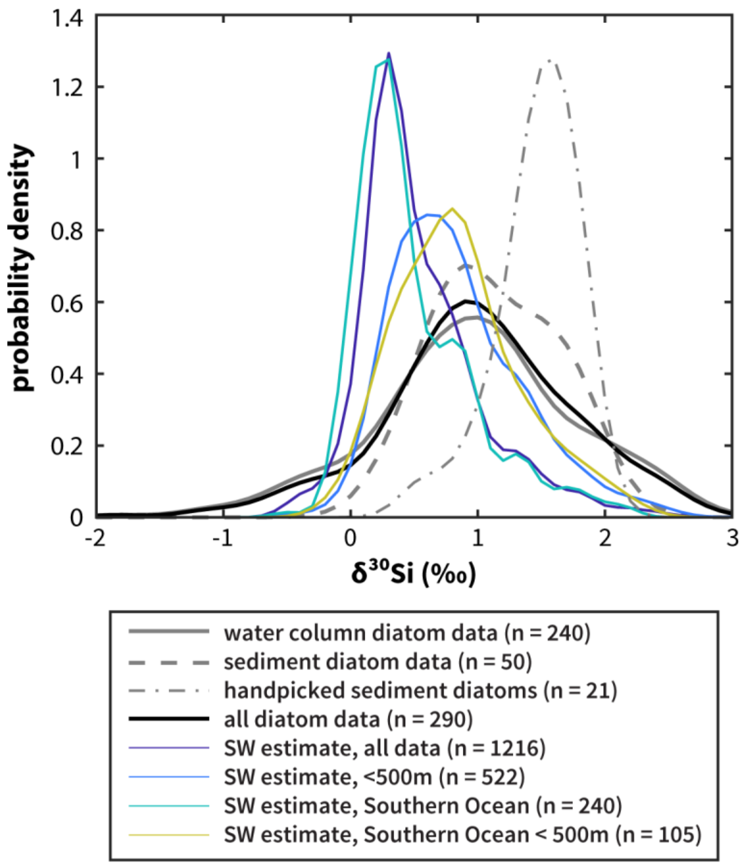

Fig. S4.

Sensitivity test of pooling all measured diatom data versus trying to estimate the diatom pool from seawater δ^{30}Si. Solid gray, dashed gray, and dash-dot gray lines show δ^{30}Si distributions of water column diatoms, sediment diatoms, and handpicked sediment diatoms, respectively, from our compilation. The sediment and water column diatom δ^{30}Si distributions are not substantially different from each other; the handpicked sediment diatoms do appear different, but this dataset is so small in number (n = 21) that it is likely less representative globally than the other two datasets. Colored lines illustrate estimates of the δ^{30}Si distribution of diatoms using seawater δ^{30}Si data from our compilation by assuming a constant -1.1‰ offset between $\delta^{30}Si_{seawater}$ and $\delta^{30}Si_{diatom}$. We compare predictions when we use all seawater δ^{30}Si data, just data from surface seawater (<500 m depth), just data from the Southern Ocean where most diatom productivity occurs, and just surface seawater from the Southern Ocean. Although estimates based on Southern Ocean seawater δ^{30}Si values are the closes to the diatom data in our compilation, all estimates are biased to lower δ^{30}Si values than observations. This is the expected pattern if, instead of assuming a constant δ^{30}Si offset between seawater and diatom frustules, we must instead incorporate a Rayleigh distillation effect. However, given the substantial additional uncertainty required to incorporate this effect, we concluded that actual measurements of diatoms are more representative of this sink globally than estimates based on seawater δ^{30}Si values.

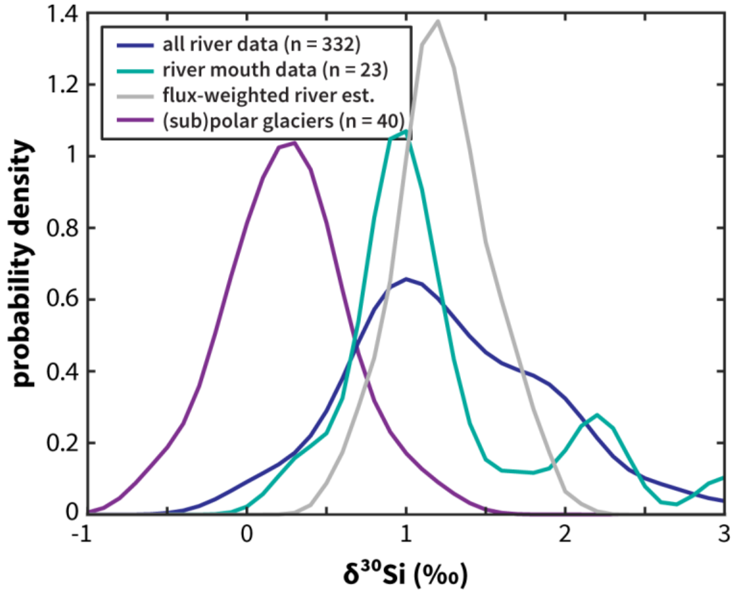

Fig. S5.

Comparison of different estimates of fluvial dSi $\delta^{30}Si$. The blue curve shows all $\delta^{30}Si$ data from rivers. The teal curve shows data only from mouths of rivers, which is, in theory, the most representative of the fluvial dSi that is actually delivered to the oceans; however, this dataset is limited by its small size (n = 23). The gray curve shows the flux-weighted estimate we developed by matching each river in our database to its catchment and weighting each suite of river $\delta^{30}Si$ values by catchment dSi flux. This represents the value we used in the model because it accounts for the fact that rivers with higher dSi fluxes influence seawater $\delta^{30}Si$ more than rivers with lower dSi fluxes. Finally, the purple curve shows the $\delta^{30}Si$ data from (sub)polar glaciers (also referred to as ice-sheet meltwaters), which are meaningfully different than the global river data.

Table S1: Summary of mean δ^{30}Si compositions of various reactive silica pools on the surface earth

Reservoir	Symbol	Mean δ^{30}Si (‰)	(n)	Median (‰)	Percentile (‰) 10th	90th	Refs[*]
**Inputs (corresponding to Table 1)**							
Rivers - dSi	$\delta^{30}Si_{River-dSi}$	1.21 ± 0.76	372	1.1	0.24	2.1	(*2*, *8*, *67–81*)
Rivers - aSi	$\delta^{30}Si_{River-aSi}$	-0.27 ± 0.30	75	-0.30	-0.68	0.10	(*77*, *79*, *81*, *82*)
Groundwater (Fresh)	$\delta^{30}Si_{FGW}$	1.33 ± 0.63	21	1.2	0.70	2.30	(*3*, *4*, *83*)
Groundwater (Marine)	$\delta^{30}Si_{MGW}$	1.69 ± 0.95	10	1.54	0.86	3.10	(*3, 4*)
Dust[b]	$\delta^{30}Si_{eolian}$	-1.03 ± 0.70	194	-0.92	-2	-0.22	(*84–90*)
LT weathering of siliceous marine sediment	$\delta^{30}Si_{W}$	-0.3[c]					
Hydro-thermal vent fluid	$\delta^{30}Si_{HT}$	-0.3 ± 0.14	2				(*8*)
Ice Sheet Meltwaters	$\delta^{30}Si_{ISM}$	0.25 ± 0.37	40	0.24	-0.14	0.65	(*73*, *79*, *82*)
**Outputs (corresponding to Table 1)**							
Diatoms in seabed	$\delta^{30}Si_{BDiatom}$[a]	1.13 ± 0.44	50	1.02	0.58	1.74	(*43*, *67*, *91*, *92*)
Sponges	$\delta^{30}Si_{Sponge}$	-1.95 ± 1.48	72	-2.14	-3.69	0.09	(*93–96*)
**Other reactive Si pools**							

Pelagic diatoms	$\delta^{30}Si_{PDiatom}$[a]	0.97	±	0.79	240	0.98	-0.063	2.02	(*67*, *93*, *97–101*)
Seawater	$\delta^{30}Si_{SW}$	1.67	±	0.45	1426	1.61	1.22	2.28	(*2*, *8*, *67*, *97*, *102–110*)
Marine porewater	$\delta^{30}Si_{PW}$	1.42	±	0.33	138	1.39	1.09	1.84	(*5*, *43*, *111*)
Radiolarians	$\delta^{30}Si_{Radiolaria}$	1.12	±	0.40	38	1.12	0.66	1.65	(*112*, *113*)
Pedogenic clays	$\delta^{30}Si_{PC}$	-1.28	±	0.70	104	-1.15	-2.27	-0.47	(*6*, *84*, *87*, *89*, *90*, *94*, *114-117*)
Phytoliths	$\delta^{30}Si_{phytolith}$	0.06	±	1.35	216	-0.10	-1.40	1.68	(*64*, *76*, *84*, *90*, *94*, *115*, *118–125*)
Freshwater diatoms	$\delta^{30}Si_{FWDiatom}$	1.14	±	0.73	33	1.22	0.21	1.92	(*80*, *126–128*)

*Refs=references

[a]All diatom data are pooled into a combined distribution in the model referred to later in the text as $\delta^{30}Si_{Diatom}$

[b]This dataset includes both measurements of loess (n = 14) and measurements of soil (n = 180); the measurements of soil are heavily weighted towards δ^{30}Si values of pedogenic clay minerals rather than detrital primary silicate minerals. We therefore chose to use only the measurements of loess (mean –0.22 ± 0.03‰, mean –0.22‰, 10th percentile –0.26‰, 90th percentile –0.19‰) in our model. The distribution of values illustrated in Figure 1A represents the loess-only dataset.

[c]Low temperature dissolution of siliceous marine sediment will be comprised of a (1) contribution from dissolution of deposited river aSi along the seabed and (2) a contribution from basaltic glass dissolution. We predict both contributions will have a signature like that of river aSi.

REFERENCES

1. P. Molnar, P. England, Late Cenozoic uplift of mountain ranges and global climate change: Chicken or egg? **346**, 29–34 (1990).

2. Z. Zhang, Z. Cao, P. Grasse, M. Dai, L. Gao, H. Kuhnert, M. Gledhill, C. M. Chiessi, K. Doering, M. Frank, Dissolved silicon isotope dynamics in large river estuaries. *Geochim. Cosmochim. Acta* **273**, 367–382 (2020).

3. C. Ehlert, A. Reckhardt, J. Greskowiak, B. T. P. Liguori, P. Böning, R. Paffrath, H. J. Brumsack, K. Pahnke, Transformation of silicon in a sandy beach ecosystem: Insights from stable silicon isotopes from fresh and saline groundwaters. *Chem. Geol.* **440**, 207–218 (2016).

4. A. N. Martin, K. Meredith, A. Baker, M. D. Norman, E. Bryan, The evolution of stable silicon isotopes in a coastal carbonate aquifer on Rottnest Island, Western Australia. *Hydrol. Earth Syst. Sci.* **25**, 3837–3853 (2021).

5. C. Ehlert, K. Doering, K. Wallmann, F. Scholz, S. Sommer, P. Grasse, S. Geilert, M. Frank, Stable silicon isotope signatures of marine pore waters—Biogenic opal dissolution versus authigenic clay mineral formation. *Geochim. Cosmochim. Acta* **191**, 102–117 (2016).

6. J. Cornelis, D. Weis, L. Lavkulich, M. Vermeire, B. Delvaux, J. Barling, Silicon isotopes record dissolution and re-precipitation of pedogenic clay minerals in a podzolic soil chronosequence. *Geoderma* **235-236**, 19–29 (2014).

7. P. J. Frings, W. Clymans, G. Fontorbe, C. L. De La Rocha, D. J. Conley, The continental Si cycle and its impact on the ocean Si isotope budget. *Chem. Geol.* **425**, 12–36 (2016).

8. C. L. De La Rocha, M. A. Brzezinski, M. J. Deniro, A first look at the distribution of the stable isotopes of silicon in natural waters. *Geochim. Cosmochim. Acta* **64**, 2467–2477 (2000).

9. J. Marin-Carbonne, M. Chaussidon, F. Robert, Micrometer-scale chemical and isotopic criteria (O and Si) on the origin and history of Precambrian cherts: Implications for paleo-temperature reconstructions. *Geochim. Cosmochim. Acta* **92**, 129–147 (2012).

10. S. Geilert, P. Grasse, K. Wallmann, V. Liebetrau, C. D. Menzies, Serpentine alteration as source of high dissolved silicon and elevated δ^{30}Si values to the marine Si cycle. *Nat. Commun.* **11**, 5123 (2020).

11. J. N. Sutton, L. André, D. Cardinal, D. J. Conley, G. F. de Souza, J. Dean, J. Dodd, C. Ehlert, M. J. Ellwood, P. J. Frings, P. Grasse, K. Hendry, M. J. Leng, P. Michalopoulos, V. N. Panizzo, G. E. A. Swann, A review of the stable isotope bio-geochemistry of the global silicon cycle and its associated trace elements. *Front. Earth Sci.* **5**, 112 (2018).

12. P. J. Tréguer, J. N. Sutton, M. Brzezinski, M. A. Charette, T. Devries, S. Dutkiewicz, C. Ehlert, J. Hawkings, A. Leynaert, S. M. Liu, N. L. Monferrer, M. López-Acosta, M. Maldonado, S. Rahman, L. Ran, O. J. Rouxel, Reviews and syntheses: The biogeochemical cycle of silicon in the modern ocean. *Biogeosciences* **18**, 1269–1289 (2021).

13. J. C. G. Walker, P. B. Hays, J. F. Kasting, A negative feedback mechanism for the long-term stabilization of Earth's surface temperature. *J. Geophys. Res.* **86**, 9776–9782 (1981).

14. R. A. Berner, A. C. Lasaga, R. M. Garrels, The carbonate-silicate geochemical cycle and its effect on atmospheric carbon dioxide over the past 100 million years. *Am. J. Sci.* **283**, 641–683 (1983).

15. Y. Park, P. Maffre, Y. GoddérisGodd, F. A. Macdonald, E. S. C Anttila, N. L. Swanson-Hysell, Emergence of the Southeast Asian islands as a driver for Neogene cooling. **13**, 25319–25326 (2011).

16. R. M. Garrels, R. A. Berner, "The global carbonate-silicate sedimentary system—Some feedback relations" in *Biomineralization and Biological Metal Accumulation: Biological and Geological Perspectives Papers presented at the Fourth International Symposium on Biomineralization, Renesse, The Netherlands, June 2–5, 1982* (Springer Netherlands, 1983), pp. 73–87.

17. D. E. Ibarra, J. K. Caves Rugenstein, A. Bachan, A. Baresch, K. V. Lau, D. L. Thomas, J. E. Lee, C. Kevin Boyce, C. Page Chamberlain, Modeling the consequences of land plant evolution on silicate weathering. *Am. J. Sci.* **319**, 1–43 (2019).

18. L. R. Kump, S. L. Brantley, Chemical weathering, atmospheric CO_2, and climate. *Annu. Rev. Earth Planet. Sci.* **28**, 611–667 (2000).

19. J. A. West, Thickness of the chemical weathering zone and implications for erosional and climatic drivers of weathering and for carbon-cycle feedbacks. *Geology* **40**, 811–814 (2012).

20. K. Maher, C. P. Chamberlain, Hydrologic regulation of chemical weathering and the geologic. *Science* **343**, 1502–1504 (2014).

21. D. J. DeMaster, The diagenesis of biogenic silica: Chemical transformations occurring in the water column, seabed, and crust. *Treatise Geochemistry.* **7**, 87–98 (2003).

22. P. Michalopoulos, R. C. Aller, Rapid clay mineral formation in amazon delta sediments - reverse weathering and oceanic elemental cycles. *Science* **270**, 614–617 (1995).

23. P. Michalopoulos, R. C. Aller, Early diagenesis of biogenic silica in the Amazon delta: Alteration, authigenic clay formation, and storage. *Geochim. Cosmochim. Acta* **68**, 1061–1085 (2004).

24. S. Rahman, R. C. Aller, J. K. Cochran, The missing silica sink: Revisiting the marine sedimentary si cycle using cosmogenic ^{32}Si. *Global Biogeochem. Cycles* **31**, 1559–1578 (2017).

25. S. Rahman, R. C. Aller, J. K. Cochran, Cosmogenic ^{32}Si as a tracer of biogenic silica burial and diagenesis: Major deltaic sinks in the silica cycle. *Geophys. Res. Lett.* **43**, 7124–7132 (2016).

26. S. Rahman, Reverse weathering reactions in marine sediments, in *Encyclopedia of Ocean Sciences* (2019), pp. 216–227.

27. F. T. Mackenzie, R. M. Garrels, Silica-bicarbonate balance in the ocean and early diagenesis. *J. Sediment. Res.* **36**, 1075–1084 (1966).

28. A. G. Dunlea, R. W. Murray, D. P. Santiago Ramos, J. A. Higgins, Cenozoic global cooling and increased seawater Mg/Ca via reduced reverse weathering. *Nat. Commun.* **8**, 844 (2017).

29. T. T. Isson, N. J. Planavsky, Reverse weathering as a long-term stabilizer of marine pH and planetary climate. *Nature* **560**, 471–475 (2018).

30. J. Krissansen-Totton, D. C. Catling, A coupled carbon-silicon cycle model over Earth history: Reverse weathering as a possible explanation of a warm mid-Proterozoic climate. *Earth Planet Sci. Lett.* **537**, 116181 (2020).

31. L. G. Sillen, The physical chemistry of seawater in *Oceanography,* M. Sears, Ed. (AAAS, Washington, D.C., 1961), p. 549.

32. F. T. Mackenzie, B. Ristvet, D. C. Thorstenson, A. Lerman, R. H. Leeper, Reverse weathering and chemical mass balance in a coastal environment, in River Inputs to Ocean Systems, J. M. Marten, J. D. Burton, D. Eisma, Eds. (UNEP and UNESCO, Switzerland, 1981), pp. 152–187.

33. B. L. Ristvet, *Reverse Weathering Reactions Within Recent Nearshore Marine Sediments, Kaneohe Bay, Oahu* (1978).

34. P. J. Tréguer, C. L. De La Rocha, The world ocean silica cycle. *Ann. Rev. Mar. Sci.* **5**, 477–501 (2013).

35. H. M. Cho, G. Kim, E. Y. Kwon, N. Moosdorf, J. Garcia-Orellana, I. R. Santos, Radium tracing nutrient inputs through submarine groundwater discharge in the global ocean. *Sci. Rep.* **8**, 4–10 (2018).

36. S. Rahman, J. J. Tamborski, M. A. Charette, J. K. Cochran, Dissolved silica in the subterranean estuary and the impact of submarine groundwater discharge on the global marine silica budget. *Mar. Chem.* **208**, 29–42 (2019).

37. M. Maldonado, M. López-Acosta, C. Sitjà, M. García-Puig, C. Galobart, G. Ercilla, A. Leynaert, Sponge skeletons as an important sink of silicon in the global oceans. *Nat. Geosci.* **12**, 815–822 (2019).

38. E. J. Trower, W. W. Fischer, Precambrian Si isotope mass balance, weathering, and the significance of the authigenic clay silica sink. *Sediment Geol.* **384**, 1–11 (2019).

39. S. P. Singh, S. K. Singh, R. Bhushan, V. K. Rai, Dissolved silicon and its isotopes in the water column of the Bay of Bengal: Internal cycling versus lateral transport. *Geochim. Cosmochim. Acta* **151**, 172–191 (2015).

40. G. G. Laruelle, V. Roubeix, A. Sferratore, B. Brodherr, D. Ciuffa, D. J. Conley, H. H. Dürr, J. Garnier, C. Lancelot, Q. LeThiPhuong, J. D. Meunier, M. Meybeck, P. Michalopoulos, B. Moriceau, S. Ni Longphuirt, S. Loucaides, L. Papush, M. Presti, O. Ragueneau, P. Regnier, L. Saccone, C. P. Slomp, C. Spiteri, P. Van Cappellen, Anthropogenic perturbations of the silicon cycle at the global scale: Key role of the land-ocean transition. *Global Biogeochem. Cycles* **23**, 1–17 (2009).

41. P. Tréguer, D. M. Nelson, A. J. Van Bennekom, D. J. DeMaster, A. Leynaert, B. Queguiner, The silica balance in the world ocean: A reestimate. *Science* **268**, 375–379 (1995).

42. L. S. Chong, W. M. Berelson, J. McManus, D. E. Hammond, N. E. Rollins, P. L. Yager, Carbon and biogenic silica export influenced by the Amazon River Plume: Patterns of remineralization in deep-sea sediments. *Deep Sea Res. 1 Oceanogr. Res. Pap.* **85**, 124–137 (2014).

43. S. Geilert, P. Grasse, K. Doering, K. Wallmann, C. Ehlert, F. Scholz, M. Frank, M. Schmidt, C. Hensen, Impact of ambient conditions on the Si isotope fractionation in marine pore fluids during early diagenesis. *Biogeosciences.* **17**, 1745–1763 (2020).

44. A. Baldermann, L. N. Warr, I. Letofsky-Papst, V. Mavromatis, Substantial iron sequestration during green-clay authigenesis in modern deep-sea sediments. *Nat. Geosci.* **8**, 885–889 (2015).

45. M. Luo, W. Li, S. Geilert, A. W. Dale, Z. Song, D. Chen, Active silica diagenesis in the deepest hadal trench sediments. *Geophys. Res. Lett.* **49**, e2022GL099365 (2022).

46. C. George, W. S. Moore, S. M. White, E. Smoak, S. B. Joye, A. Leier, A. M. Wilson, A new mechanism for submarine groundwater discharge from continental shelves. *Water Resour. Res.* **56**, 1–17 (2020).

47. C. L. De La Rocha, M. A. Brzezinski, M. J. DeNiro, Fractionation of silicon isotopes by marine diatoms during biogenic silica formation. *Geochim. Cosmochim. Acta* **61**, 5051–5056 (1997).

48. Y. Huh, L.-H. Chan, L. Zhang, J. M. Edmond, Lithium and its isotopes in major world rivers: Implications for weathering and the oceanic budget. *Geochim. Cosmochim. Acta* **62**, 2039–2051 (1998).

49. J. S. Pistiner, G. M. Henderson, Lithium-isotope fractionation during continental weathering processes. *Earth Planet. Sci. Lett.* **214**, 327–339 (2003).

50. S. Penniston-Dorland, X. M. Liu, R. L. Rudnick, Lithium isotope geochemistry. *Non-Traditional Stable Isotopes.* **82**, 165–218 (2017).

51. G. Li, A. J. West, Evolution of Cenozoic seawater lithium isotopes: Coupling of global denudation regime and shifting seawater sinks. *Earth Planet. Sci. Lett.* **401**, 284–293 (2014).

52. S. Misra, P. N. Froelich, Lithium isotope history of Cenozoic seawater: Changes in silicate weathering and reverse weathering. *Science* **335**, 818–823 (2012).

53. E. C. Hathorne, R. H. James, Temporal record of lithium in seawater: A tracer for silicate weathering? *Earth Planet. Sci. Lett.* **246**, 393–406 (2006).

54. M. J. Murphy, D. Porcelli, P. A. E. Pogge von Strandmann, C. A. Hirst, L. Kutscher, J. A. Katchinoff, C. M. Mörth, T. Maximov, P. S. Andersson, Tracing silicate weathering processes in the permafrost-dominated Lena River watershed using lithium isotopes. *Geochim. Cosmochim. Acta* **245**, 154–171 (2019).

55. R. Dupuis, M. Benoit, M. E. Tuckerman, M. Méheut, Importance of a fully anharmonic treatment of equilibrium isotope fractionation properties of dissolved ionic species as evidenced by Li^+(aq). *Acc. Chem. Res.* **50**, 1597–1605 (2017).

56. R. S. Hindshaw, R. Tosca, T. L. Goût, I. Farnan, N. J. Tosca, E. T. Tipper, Experimental constraints on Li isotope fractionation during clay formation. *Geochim. Cosmochim. Acta* **250**, 219–237 (2019).

57. N. Vigier, A. Decarreau, R. Millot, J. Carignan, S. Petit, C. France-Lanord, Quantifying Li isotope fractionation during smectite formation and implications for the Li cycle. *Geochim. Cosmochim. Acta* **72**, 780–792 (2008).

58. P. A. Suchet, J.-L. Probst, W. Ludwig, Worldwide distribution of continental rock lithology: Implications for the atmospheric/soil CO_2 uptake by continental weathering and alkalinity river transport to the oceans. *Global Biogeochem. Cycles* **17**, 1038 (2003).

59. A. Alexandre, J.-D. Meunier, F. Colin, J.-M. Koud, Plant impact on the biogeochemical cycle of silicon and related weathering processes. *Geochim. Cosmochim. Acta* **61**, 677–682 (1997).

60. D. J. Conley, Riverine contribution of biogenic silica to the oceanic silica budget. *Limnol. Oceanogr.* **42**, 774–777 (1997).

61. A. Zhang, J. Zhang, S. Liu, Spatial and temporal variations of dissolved silicon isotope compositions in a large dammed river system. *Chem. Geol.* **545**, 119645 (2020).

62. M. Meybeck, H. H. Dürr, C. J. Vörösmarty, Global coastal segmentation and its river catchment contributors: A new look at land-ocean linkage. *Global Biogeochem. Cycles* **20**, GB1S90 (2006).

63. H. H. Dürr, M. Meybeck, J. Hartmann, G. G. Laruelle, V. Roubeix, Global spatial distribution of natural riverine silica inputs to the coastal zone. *Biogeosciences.* **8**, 597–620 (2011).

64. K. Ziegler, O. A. Hadwick, M. Brzezinski, E. F. Kelly, Natural variations of δ30Si ratios during progressive basalt weathering, Hawaiian Islands. *Geochim. Cosmochim. Acta* **69**, 4597–4610 (2005).

65. D. J. DeMaster, The accumulation and cycling of biogenic silica in the Southern Ocean: Revisiting the marine silica budget. *Deep Sea Res. 2 Top Stud. Oceanogr.* **49**, 3155–3167 (2002).

66. D. J. DeMaster, *The global marine silica budget: Sources and sinks* (Elsevier Ltd., ed. 3, 2019; http://dx.doi.org/10.1016/B978-0-12-409548-9.10799-7).

67. Z. Cao, M. Frank, M. Dai, P. Grasse, C. Ehlert, Silicon isotope constraints on sources and utilization of silicic acid in the northern South China Sea. *Geochim. Cosmochim. Acta* **97**, 88–104 (2012).

68. H. E. Cockerton, F. A. Street-Perrott, M. J. Leng, P. A. Barker, M. S. A. Horstwood, V. Pashley, Stable-isotope (H, O, and Si) evidence for seasonal variations in hydrology and Si cycling from modern waters in the Nile Basin: Implications for interpreting the quaternary record. *Quat Sci Rev.* **66**, 4–21 (2013).

69. H. J. Hughes, S. Bouillon, L. André, D. Cardinal, The effects of weathering variability and anthropogenic pressures upon silicon cycling in an intertropical watershed (Tana River, Kenya). *Chem. Geol.* **308-309**, 18–25 (2012).

70. D. Cardinal, J. Gaillardet, H. J. Hughes, S. Opfergelt, L. André, Contrasting silicon isotope signatures in rivers from the Congo Basin and the specific behaviour of organic-rich waters. *Geophys. Res. Lett.* **37**, 1–6 (2010).

71. H. J. Hughes, F. Sondag, C. Cocquyt, A. Laraque, A. Pandi, L. André, D. Cardinala, Effect of seasonal biogenic silica variations on dissolved silicon fluxes and isotopic signatures in the Congo River. *Limnol. Oceanogr.* **56**, 551–561 (2011).

72. C. Delvaux, D. Cardinal, V. Carbonnel, L. Chou, H. J. Hughes, L. André, Controls on riverine δ30Si signatures in a temperate watershed under high anthropogenic pressure (Scheldt - Belgium). *J. Mar. Syst.* **128**, 40–51 (2013).

73. S. Opfergelt, K. W. Burton, P. A. E. Pogge von Strandmann, S. R. Gislason, A. N. Halliday, Riverine silicon isotope variations in glaciated basaltic terrains: Implications for the Si delivery to the ocean over glacial-interglacial intervals. *Earth Planet. Sci. Lett.* **369-370**, 211–219 (2013).

74. R. B. Georg, B. C. Reynolds, A. J. West, K. W. Burton, A. N. Halliday, Silicon isotope variations accompanying basalt weathering in Iceland. *Earth Planet. Sci. Lett.* **261**, 476–490 (2007).

75. H. J. Hughes, F. Sondag, R. V. Santos, L. André, D. Cardinal, The riverine silicon isotope composition of the Amazon Basin. *Geochim. Cosmochim. Acta* **121**, 637–651 (2013).

76. O. S. Pokrovsky, B. C. Reynolds, A. S. Prokushkin, J. Schott, J. Viers, Silicon isotope variations in Central Siberian rivers during basalt weathering in permafrost-dominated larch forests. *Chem. Geol.* **355**, 103–116 (2013).

77. T. P. Ding, J. F. Gao, S. H. Tian, H. B. Wang, M. Li, Silicon isotopic composition of dissolved silicon and suspended particulate matter in the Yellow River, China, with implications for the global silicon cycle. *Geochim. Cosmochim. Acta* **75**, 6672–6689 (2011).

78. P. J. Frings, W. Clymans, G. Fontorbe, W. Gray, G. Chakrapani, D. J. Conley, C. De La Rocha, Silicate weathering in the Ganges alluvial plain. *Earth Planet. Sci. Lett.* **427**, 136–148 (2015).

79. H. V. Pryer, J. E. Hatton, J. L. Wadham, J. R. Hawkings, L. F. Robinson, A. M. Kellerman, M. G. Marshall, A. Urra, A. Covey, G. Daneri, V. Häussermann, K. R. Hendry, The effects of glacial cover on riverine silicon isotope compositions in chilean patagonia. *Front. Earth Sci. (Lausanne).* **8**, (2020). 10.3389/feart.2020.00368

80. L. Y. Alleman, D. Cardinal, C. Cocquyt, P. D. Plisnier, J. P. Descy, I. Kimirei, D. Sinyinza, L. André, Silicon isotopic fractionation in Lake Tanganyika and its main tributaries. *J. Great Lakes Res.* **31**, 509–519 (2005).

81. T. Ding, D. Wan, C. Wang, F. Zhang, Silicon isotope compositions of dissolved silicon and suspended matter in the Yangtze River, China. *Geochim. Cosmochim. Acta* **68**, 205–216 (2004).

82. J. E. Hatton, K. R. Hendry, J. R. Hawkings, J. L. Wadham, S. Opfergelt, T. J. Kohler, J. C. Yde, M. Stibal, J. D. Žárský, Silicon isotopes in Arctic and sub-Arctic glacial meltwaters: The role of subglacial weathering in the silicon cycle. *Proc. Math. Phys. Eng. Sci.* **475**, 20190098 (2019).

83. R. B. Georg, A. J. West, A. R. Basu, A. N. Halliday, Silicon fluxes and isotope composition of direct groundwater discharge into the Bay of Bengal and the effect on the global ocean silicon isotope budget. *Earth Planet. Sci. Lett.* **283**, 67–74 (2009).

84. J. T. Cornelis, J. Ranger, A. Iserentant, B. Delvaux, Tree species impact the terrestrial cycle of silicon through various uptakes. *Biogeochemistry* **97**, 231–245 (2010).

85. P. J. Frings, C. De La Rocha, E. Struyf, D. van Pelt, J. Schoelynck, M. M. Hudson, M. J. Gondwe, P. Wolski, K. Mosimane, W. Gray, J. Schaller, D. J. Conley, Tracing silicon cycling in the Okavango Delta, a sub-tropical flood-pulse wetland using silicon isotopes. *Geochim. Cosmochim. Acta* **142**, 132–148 (2014).

86. P. S. Savage, R. B. Georg, H. M. Williams, A. N. Halliday, The silicon isotope composition of the upper continental crust. *Geochim. Cosmochim. Acta* **109**, 384–399 (2013).

87. C. R. Bern, M. A. Brzezinski, C. Beucher, K. Ziegler, O. A. Chadwick, Weathering, dust, and biocycling effects on soil silicon isotope ratios. *Geochim. Cosmochim. Acta* **74**, 876–889 (2010).

88. P. A. E. Pogge von Strandmann, S. Opfergelt, Y. J. Lai, B. Sigfússon, S. R. Gislason, K. W. Burton, Lithium, magnesium and silicon isotope behaviour accompanying weathering in a basaltic soil and pore water profile in Iceland. *Earth Planet. Sci. Lett.* **339-340**, 11–23 (2012).

89. S. Opfergelt, R. B. Georg, B. Delvaux, Y. M. Cabidoche, K. W. Burton, A. N. Halliday, Silicon isotopes and the tracing of desilication in volcanic soil weathering sequences, Guadeloupe. *Chem Geol.* **326-327**, 113–122 (2012).

90. G. Steinhoefel, J. Breuer, F. von Blanckenburg, I. Horn, D. Kaczorek, M. Sommer, Micrometer silicon isotope diagnostics of soils by UV femtosecond laser ablation. *Chem. Geol.* **286**, 280–289 (2011).

91. R. A. Pickering, L. Cassarino, K. R. Hendry, X. L. Wang, K. Maiti, J. W. Krause, Using stable isotopes to disentangle marine sedimentary signals in reactive silicon pools. *Geophys. Res. Lett.* **47**, e2020GL087877 (2020).

92. K. Doering, C. Ehlert, P. Grasse, X. Crosta, S. Fleury, M. Frank, R. Schneider, Differences between mono-generic and mixed diatom silicon isotope compositions trace present and past nutrient utilisation off Peru. *Geochim. Cosmochim. Acta* **177**, 30–47 (2016).

93. K. R. Hendry, L. F. Robinson, The relationship between silicon isotope fractionation in sponges and silicic acid concentration: Modern and core-top studies of biogenic opal. *Geochim. Cosmochim. Acta* **81**, 1–12 (2012).

94. C. B. Douthitt, The geochemistry of the stable isotopes of silicon. *Geochim. Cosmochim. Acta* **46**, 1449–1485 (1982).

95. C. L. De la Rocha, Silicon isotope fractionation by marine sponges and the reconstruction of the silicon isotope composition of ancient deep water. *Geology* **31**, 423–426 (2003).

96. M. Wille, J. Sutton, M. J. Ellwood, M. Sambridge, W. Maher, S. Eggins, M. Kelly, Silicon isotopic fractionation in marine sponges: A new model for understanding silicon isotopic variations in sponges. *Earth Planet. Sci. Lett.* **292**, 281–289 (2010).

97. F. Fripiat, A. J. Cavagna, F. Dehairs, S. Speich, L. André, D. Cardinal, Silicon pool dynamics and biogenic silica export in the Southern Ocean inferred from Si-isotopes. *Ocean Science* **7**, 533–547 (2011).

98. D. Cardinal, N. Savoye, T. W. Trull, F. Dehairs, E. E. Kopczynska, F. Fripiat, J. L. Tison, L. André, Silicon isotopes in spring Southern Ocean diatoms: Large zonal changes despite homogeneity among size fractions. *Mar. Chem.* **106**, 46–62 (2007).

99. K. E. Egan, R. E. M. Rickaby, M. J. Leng, K. R. Hendry, M. Hermoso, H. J. Sloane, H. Bostock, A. N. Halliday, Diatom silicon isotopes as a proxy for silicic acid utilisation: A Southern Ocean core top calibration. *Geochim. Cosmochim. Acta* **96**, 174–192 (2012).

100. D. E. Varela, C. J. Pride, M. A. Brzezinski, Biological fractionation of silicon isotopes in Southern Ocean surface waters. *Global Biogeochem. Cycles* **18**, 1–8 (2004).

101. F. Fripiat, A. J. Cavagna, F. Dehairs, A. De Brauwere, L. André, D. Cardinal, Processes controlling the Si-isotopic composition in the Southern Ocean and application for paleoceanography. *Biogeosciences* **9**, 2443–2457 (2012).

102. D. Cardinal, L. Y. Alleman, F. Dehairs, N. Savoye, T. W. Trull, L. André, Relevance of silicon isotopes to Si-nutrient utilization and Si-source assessment in Antarctic waters. *Global Biogeochem. Cycles* **19**, GB2007 (2005).

103. G. F. De Souza, B. C. Reynolds, J. Rickli, M. Frank, M. A. Saito, L. J. A. Gerringa, B. Bourdon, Southern Ocean control of silicon stable isotope distribution in the deep Atlantic Ocean. *Global Biogeochem. Cycles* **26**, 1–13 (2012).

104. M. A. Brzezinski, J. L. Jones, Coupling of the distribution of silicon isotopes to the meridional overturning circulation of the North Atlantic Ocean. *Deep Sea Res. 2 Top Stud. Oceanogr.* **116**, 79–88 (2015).

105. J. N. Sutton, G. F. De Souza, M. I. García-Ibáñez, C. L. D. La Rocha, The silicon stable isotope distribution along the GEOVIDE section (GEOTRACES GA-01) of the North Atlantic Ocean. *Biogeosciences* **15**, 5663–5676 (2018).

106. P. Grasse, C. Ehlert, M. Frank, The influence of water mass mixing on the dissolved Si isotope composition in the Eastern Equatorial Pacific. *Earth Planet. Sci. Lett.* **380**, 60–71 (2013).

107. P. Grasse, E. Ryabenko, C. Ehlert, M. A. Altabet, M. Frank, Silicon and nitrogen cycling in the upwelling area off Peru: A dual isotope approach. *Limnol. Oceanogr.* **61**, 1661–1676 (2016).

108. B. C. Reynolds, M. Frank, A. N. Halliday, Silicon isotope fractionation during nutrient utilization in the North Pacific. *Earth Planet Sci. Lett.* **244**, 431–443 (2006).

109. D. E. Varela, M. A. Brzezinski, C. P. Beucher, J. L. Jones, K. E. Giesbrecht, B. Lansard, A. Mucci, Heavy silicon isotopic composition of silicic acid and biogenic silica in Arctic waters over the Beaufort shelf and the Canada Basin. *Global Biogeochem. Cycles* **30**, 804–824 (2016).

110. C. P. Beucher, M. A. Brzezinski, J. L. Jones, Mechanisms controlling silicon isotope distribution in the Eastern Equatorial Pacific. *Geochim. Cosmochim. Acta* **75**, 4286–4294 (2011).

111. H. C. Ng, L. Cassarino, R. A. Pickering, E. M. S. Woodward, S. J. Hammond, K. R. Hendry, Sediment efflux of silicon on the Greenland margin and implications for the marine silicon cycle. *Earth Planet. Sci. Lett.* **529**, 115877 (2020).

112. A. Abelmann, R. Gersonde, G. Knorr, X. Zhang, B. Chapligin, E. Maier, O. Esper, H. Friedrichsen, G. Lohmann, H. Meyer, R. Tiedemann, The seasonal sea-ice zone in the glacial Southern Ocean as a carbon sink. *Nat. Commun.* **6**, 8136 (2015).

113. K. Doering, C. Ehlert, K. Pahnke, M. Frank, R. Schneider, P. Grasse, Silicon isotope signatures of radiolaria reveal taxon-specific differences in isotope fractionation. *Front. Mar. Sci.* **8**, 666896 (2021).

114. K. Ziegler, O. A. Chadwick, A. F. White, M. A. Brzezinski, δ^{30}Si systematics in a granitic saprolite Puerto Rico. *Geology* **33**, 817–820 (2005).

115. S. Opfergelt, B. Delvaux, L. André, D. Cardinal, Plant silicon isotopic signature might reflect soil weathering degree. *Biogeochemistry.* **91**, 163–175 (2008).

116. S. Opfergelt, D. Cardinal, L. André, C. Delvigne, L. Bremond, B. Delvaux, Variations of δ^{30}Si and Ge/Si with weathering and biogenic input in tropical basaltic ash soils under monoculture. *Geochim. Cosmochim. Acta* **74**, 225–240 (2010).

117. G. Bayon, C. Delvigne, E. Ponzevera, A. V. Borges, F. Darchambeau, P. De Deckker, T. Lambert, L. Monin, S. Toucanne, L. André, The silicon isotopic composition of fine-grained river sediments and its relation to climate and lithology. *Geochim. Cosmochim. Acta* **229**, 147–161 (2018).

118. P. J. Frings, W. Clymans, D. J. Conley, Amorphous silica transport in the Ganges basin: Implications for Si delivery to the oceans. *Proc. Earth Planet. Sci.* **10**, 271–274 (2014).

119. M. J. Hodson, A. G. Parker, M. J. Leng, H. J. Sloane, Silicon, oxygen and carbon isotope composition of wheat (*Triticum aestivum* L.) phytoliths: Implications for palaeoecology and archaeology. *J. Quat. Sci.* **23**, 331–339 (2008).

120. A. F. White, D. V. Vivit, M. S. Schulz, T. D. Bullen, R. R. Evett, J. Aagarwal, Biogenic and pedogenic controls on Si distributions and cycling in grasslands of the Santa Cruz soil chronosequence, California. *Geochim. Cosmochim. Acta* **94**, 72–94 (2012).

121. E. Engström, I. Rodushkin, B. Öhlander, J. Ingri, D. C. Baxter, Silicon isotopic composition of boreal forest vegetation in Northern Sweden. *Chem. Geol.* **257**, 247–256 (2008).

122. T. P. Ding, G. R. Ma, M. X. Shui, D. F. Wan, R. H. Li, Silicon isotope study on rice plants from the Zhejiang province, China. *Chem Geol.* **218**, 41–50 (2005).

123. T. P. Ding, J. X. Zhou, D. F. Wan, Z. Y. Chen, C. Y. Wang, F. Zhang, Silicon isotope fractionation in bamboo and its significance to the biogeochemical cycle of silicon. *Geochim. Cosmochim. Acta* **72**, 1381–1395 (2008).

124. T. P. Ding, S. H. Tian, L. Sun, L. H. Wu, J. X. Zhou, Z. Y. Chen, Silicon isotope fractionation between rice plants and nutrient solution and its significance to the study of the silicon cycle. *Geochim. Cosmochim. Acta* **72**, 5600–5615 (2008).

125. Y. Sun, L. Wu, X. Li, L. Sun, J. Gao, T. Ding, Y. Zhu, Silicon isotope fractionation in maize and its biogeochemical significance. *Anal. Lett.* **50**, 2475–2490 (2017).

126. S. Opfergelt, E. S. Eiriksdottir, K. W. Burton, A. Einarsson, C. Siebert, S. R. Gislason, A. N. Halliday, Quantifying the impact of freshwater diatom productivity on silicon isotopes and silicon fluxes: Lake Myvatn, Iceland. *Earth Planet Sci Lett.* **305**, 73–82 (2011).

127. X. Sun, P. S. Andersson, C. Humborg, M. Pastuszak, C. M. Mörth, Silicon isotope enrichment in diatoms during nutrient-limited blooms in a eutrophied river system. *J. Geochem. Explor.* **132**, 173–180 (2013).

128. V. N. Panizzo, G. E. A. Swann, A. W. Mackay, E. Vologina, M. Sturm, V. Pashley, M. S. A. Horstwood, Insights into the transfer of silicon isotopes into the sediment record. *Biogeosciences* **13**, 147–157 (2016).
